# Does Ideal Blood Pressure Vary by Cognitive Domain? A UK Biobank Study

**DOI:** 10.1111/jch.70129

**Published:** 2025-08-22

**Authors:** Matthew J. Lennon, Jan Willem Van Dalen, Jessica W. Lo, Anbupalam Thalamuthu, John D. Crawford, Aletta E. Schutte, Perminder S. Sachdev

**Affiliations:** ^1^ Centre For Healthy Brain Aging (CHeBA) Discipline of Psychiatry and Mental Health, Level 1, AGSM (G27), University of New South Wales Kensington New South Wales Australia; ^2^ Royal North Shore Hospital, Northern Sydney Local Health District St Leonards New South Wales Australia; ^3^ Department of Neurology, Donders Institute For Brain Cognition and Behaviour, Radboud University Medical Center Nijmegen The Netherlands; ^4^ The George Institute For Global Health Barangaroo New South Wales Australia; ^5^ School of Population Health Samuels Building, University of New South Wales Kensington New South Wales Australia; ^6^ Neuropsychiatric Institute The Euroa Centre, Prince of Wales Hospital Randwick New South Wales Australia

**Keywords:** antihypertensive, blood pressure, cognitive function, hypertension, reaction time, UK Biobank

## Abstract

High blood pressure (BP) is a risk factor for cognitive decline. Increasingly, studies have found the relationship to be nonlinear, with low BP also indicating higher risk. This UK Biobank study examines the nonlinear relationships between BP and cognitive function, including whether the relationships differ by cognitive domain. Systolic (SBP) and diastolic BP (DBP) were measured at baseline. Cognitive domains included fluid intelligence, attention, and reaction time, measured at baseline and over time. Nonlinear mixed‐effects regression models, including natural spline terms for SBP and DBP, were used to assess the relationships. Additional models evaluated interactions with age, sex, and hypertension history/antihypertensive use. There were 439 301 (mean age = 56.3, SD = 8.1, 45.1% male) included participants. Baseline SBP had significant inverted U‐shaped relationships with fluid intelligence (*p* < 0.0001), attention (*p* < 0.0001), and reaction time (*p* < 0.0001), with substantially different ideal SBPs for each domain (118, 127.5, and 150.5 mmHg, respectively). Baseline DBP had significant relationships with fluid intelligence (*p* < 0.0001) and attention (*p* < 0.0001), again with varying ideal DBPs (57.5 and 74.5 mmHg, respectively). Higher baseline SBP had a small, inverse relation with trajectories of attention during the study (*p* < 0.0001), but no relationship with trajectories of either fluid intelligence or reaction time. Older, male, and untreated hypertension subgroups had significantly poorer reaction time at lower baseline SBP and DBP (*p* < 0.0001). The relationship between BP and cognitive function is nonlinear with the three domains optimal at differing BP levels. Older persons, males, or hypertensive patients may be particularly susceptible to negative cognitive effects of low BP.

**Abbreviations**
CSE: Certificate of Secondary Education
DSS: Digit Symbol Substitution Test
GCSE: General Certificate of Secondary Education
HNC: Higher National Certificate
HND: Higher National Diploma
NVQ: National Vocational Qualification
PRS: Polygenic Risk Score
TMTA: Trail Making Test A
TMTB: Trail Making Test B

## 1 Introduction

Hypertension affects an estimated 32% of adults worldwide, including over two‐thirds of those older than 65 [1]. Higher blood pressure (BP) in mid‐life is associated with worse cognitive outcomes [2] and higher risk of dementia [3], but for the most part studies have been limited methodologically by only employing linear models or using simple three or four group categorizations that cannot capture nonlinear effects.

In the studies that have found U‐shaped relationships between BP and impaired cognition or dementia, the BPs associated with the best cognitive scores or lowest risk of dementia have varied widely, referred to herein as “ideal” BP. The Chicago Health and Aging Project [4] (*n* = 2137, mean age [SD] = 73.1 [5.9]) found that the lowest risk of Alzheimer's dementia was identified at an SBP of 138 mmHg and a DBP of 77 mmHg. In a 2023 IPD meta‐analysis [5] (*n* = 17 286, mean age [SD] = 83.5 [7.2]), the lowest dementia risk was found at an SBP of 185 mmHg.

While these aforementioned studies showed differing ideal BP for global measures of cognitive dysfunction (i.e., dementia diagnosis or MMSE), increasingly, studies are showing that the relationship between BP and cognition differs by cognitive domain. A systematic review [6] found that executive function and processing speed had a nonlinear or inverted U‐shaped relationship with BP in more studies than memory function, which for the most part had an inverse linear relationship with BP. In 2023, our group published a mendelian randomization analysis [7], examining the association between genetically predicted BP (i.e., BP Polygenic Risk Scores [PRSs]) and cognitive outcomes. Our study found that whereas fluid intelligence had a negative, approximately linear relationship with the SBP PRS, attention had a U‐shaped relationship and reaction time had a positive, approximately linear relationship [7]. Furthermore, males with low genetically predicted BP were significantly more likely to have poor reaction time, compared to females. Given that this study took a mendelian randomization approach, causal inferences could be made between BP and these cognitive outcomes. However, it was unclear how much of the results was explained by genetic pleiotropy and what the results meant clinically for in‐person, measured BP, given that PRSs are not used in practice.

The current study, utilizing the UK Biobank participant data, follows from this previous study and assesses the relationship between measured BP at baseline and cognitive domains. Using nonlinear models, it explores the ideal BP for each cognitive domain and whether this ideal BP differs by cognitive domain, age, sex, or antihypertensive status.

## 2 Methods

### 2.1 Participants

From the years 2006 to 2010, 502 633 participants (aged between 37 and 73) were recruited at 22 sites in the United Kingdom to be included in the UK Biobank study. Those with adequate clinical and demographic data were included in our study. Data for the current study were downloaded under application number 66035. This UK Biobank study has been detailed in previous papers [8] and ethics approval was given by the North West Multi‐Centre Research Ethics Committee (MREC).

### 2.2 Excluded Participants

Participants who had been diagnosed with serious neurological illnesses (including dementia) that may affect cognitive performance were removed from the analysis (see Table S1 for list of diagnoses).

### 2.3 Cognitive Outcomes

The UK Biobank includes a diverse set of cognitive tests with variable numbers of repeats over follow‐up waves. Tests were selected for inclusion if they were

1. Measured at baseline (i.e., could not only be part of the imaging cognitive battery)
2. Measured at follow‐up waves
3. Sufficiently discriminating in their range of scores (i.e., > 10 different scores)

Applying these criteria, five tests were chosen (Fluid intelligence, Reaction time, Digit Symbol Substitution Test [DSS], Trail Making Test A [TMTA], and Trail Making Test B [TMTB]) (see Table S2 for descriptions of tests). To reduce the number of dimensions, an orthogonal rotated principal component analysis was run to assess which of the cognitive tests group together (Table S3). The TMTA, TMTB, and DSS were grouped together by standardizing the three scores and taking their average to be a unified “attention score”. Thus, the three main cognitive outcomes were fluid intelligence, attention, and reaction time. These outcomes were standardized, and reaction time was inverted such that higher scores indicated better performance (see Supporting Information: Methods for additional details). Cognition was measured at baseline and in some participants at up to 3 additional points over the subsequent 14 years.

### 2.4 Measurement of Blood Pressure, Antihypertensive Use, and History of Hypertension

BP was either measured by an automated device (Omron HEM‐7015IT digital BP monitor) or manually (manual sphygmomanometer). Two BP measurements were taken with a 1‐minute interval. The mean of these two measurements was used as the baseline measure.

Antihypertensive use may modify the effect of BP on cognitive outcomes [9] and, thus, is an important covariate. However, as a dummy variable, current antihypertensive use would partly reflect an individual's history of hypertension and not the effect of the antihypertensive. As such, participants were classified by their self‐reported history of previous hypertension and their baseline antihypertensive use. There were four potential groups defined by this categorization:

1. No previous hypertension diagnosis and not taking an antihypertensive at baseline (“Healthy Control” participants)
2. No previous hypertension diagnosis and taking an antihypertensive at baseline (“Uncertain hypertension”)
3. Previous hypertension diagnosis and taking an antihypertensive at baseline (“Treated hypertension”)
4. Previous hypertension diagnosis and not taking an antihypertensive at baseline (“Untreated hypertension”)

The second group was considered anomalous and thus removed (Supporting Information: Methods). Antihypertensive medication subtypes, prevalence, and codes are detailed in Tables S4–S6. Coding of the covariates is detailed in the Supporting Information: Methods and Table S7.

### 2.5 Statistical Analysis

A number of previous studies have indicated that the relationship between BP and cognitive outcomes is nonlinear. Thus, we used nonlinear mixed‐effects regression models, employing natural splines to explore the relationships between baseline BP and cognitive outcomes. Further details on the function of natural splines are explained in the Supporting Information: Methods.

Using this method, the 95% confidence intervals for the BP associated with the best cognitive outcomes were calculated by running the analysis from 1000 bootstraps (taking a random sample each time), identifying the ideal BP for cognition in each bootstrap and computing the 2.5–97.5 centiles for the 1000 tests [5]. These intervals may be asymmetrical, and they indicate the BPs at which cognition is similar. Larger intervals indicate greater uncertainty about where their ideal performance is. The bootstrapped estimates for ideal BP for each cognitive domain were compared using pairwise‐Wilcoxon rank sum tests. Linear models were also fitted and compared with the nonlinear models using the AIC and log‐likelihood tests.

The main, fully adjusted analysis included both age and age^2^, a quadratic term for age, because cognitive decline accelerates with age, rather than occurring linearly [9]. Other covariates included sex, education, Townsend Deprivation Index, cardiovascular disease history, diabetes status, smoking status, physical activity, alcohol use frequency, BMI, and history of hypertension/antihypertensive use. Nonlinear mixed‐effects regression models were used to assess the effect of BP on cognitive scores at baseline and over time. Time in study and its interaction with the BP natural spline term were used to assess the cognitive trajectories over the study course. In order to assess moderating effects of age, sex, and antihypertensive use, models including interaction terms between these three predictors and the main natural spline terms for baseline BP were run. For the purposes of this analysis, age was categorized as a three‐level categorical variable (40–49, 50–59, 60–70), based on sample size balance, and estimates of effects in each age group were computed by centering age at each level.

Because of the complex structure of the UK Biobank and large variability in the numbers of participants who have completed each of the cognitive tests, a number of sensitivity analyses were performed.

First, to assess the robustness of the construct of the attention score, the main analysis was repeated with TMTA, TMTB, and DSS as the outcomes. Second, to minimize exclusion of participants, a partially adjusted analysis was run controlling for only age, age^2^, sex, education, and Townsend Deprivation Index. Third, to ensure that results that differed by cognitive domain were not merely due to the testing of populations that differed by size and composition, we repeated the analyses including only those who had completed all five cognitive tests at baseline. Fourth, the main analysis was repeated using pulse pressure (PP) as the main predictor, to assess for possible differential effects of related BP measurements.

For each of the terms that had a natural spline component to it, rather than inspecting the significance of each of the individual natural spline coefficients, the overall significance of the natural splines was assessed by comparing model fit of models with and without that natural spline term, as in previous publications [5]. If there was a significant difference in the model fit and a lower AIC value, the interaction term was treated as significant.

The statistical analysis was performed using the nlme and splines packages in R 4.0.3. Given that three separate outcome measures were examined, a Sidak correction was applied with an adjusted *p* value threshold of 0.017.

## 3 Results

### 3.1 Participant Characteristics

There were 439 301 participants (mean age [SD] = 56.3 [8.1]) who had sufficient data to be included in the study (i.e., at least one cognitive outcome, age, sex, Townsend Deprivation Index, Education, and Blood Pressure Measure), of whom 45.1% were male (see Table 1). Of the included participants, 5.5% had a history of diabetes and 10.3% were current smokers. There were 71% of participants who reported no previous diagnosis of hypertension and were not taking an antihypertensive, whereas 14.1% had a previous diagnosis of hypertension and were taking medication, and 12.1% had a previous diagnosis but were not taking antihypertensive medication. Of those with a diagnosis of hypertension, the average time since diagnosis was 8.7 years and the majority were only taking one antihypertensive. Those with untreated hypertension reported a significantly shorter duration of hypertension compared to those with treated hypertension (7.1 vs. 9.4 years, *p* < 0.0001) (Table S8).

**TABLE 1:** Summary of participant characteristics.

Mean age (SD) (*n* = 439 301)	56.3 (8.1)
Male participants (*n* and %) (*n* = 439 301)	198 125 (45.1%)
Mean days in study (SD) (*n* = 439 301)	378.2 (1049.3)
Education group (*n* = 439 301)^a^	1–72 196 (16.4%) 2–50 068 (11.4%) 3–24 582 (5.6%) 4–24 459 (5.6%) 5–56 515 (12.9%) 6–65 016 (14.8%) 7–146 465 (33.3%)
Mean Townsend Deprivation Index (SD) (*n* = 439 301)	−1.4 (3.1)
Diabetes history (*n* and %) (*n* = 439 298)	241 161 (5.50%)
Smoking history (*n* and %) (*n* = 437 820)^b^	1–241 624 (55.2%) 2–151 123 (34.5%) 3–45 073 (10.3%)
CVD history (*n* and %) (*n* = 438 454)	10 523 (2.40%)
Mean BMI (SD) (*n* = 437 796)	27.4 (4.8)
Hypertension history/antihypertensive use (*n* and %) (*n* = 438 450)^c^	1–311 184 (71%) 2–12 306 (2.8%) 3–61 808 (14.1%) 4–53 152 (12.1%)
Numbers of antihypertensives (*n* and %)	0–237 845 (73.5%) 1–57 441 (17.7%) 2–22 892 (7.1%) 3–4729 (1.5%) 4–702 (0.2%) 5–61 (0.0%) 6–6 (0.0%) 7–1 (0.0%)
Type of antihypertensives (*N* (%))	
Angiotensin II converting enzyme inhibitors	41 131 (13.1%)
Angiotensin receptor blockers	16 315 (5.2%)
Calcium channel blockers	25 731 (8.2%)
Beta‐blockers	25 208 (8%)
Alpha 1 blockers	716 (0.2%)
Other	832 (0.3%)
Frusemide	3521 (1.1%)
Spironolactone	710 (0.2%)
Thiazides	2186 (0.7%)
Current alcohol use (*n* = 439 297)^d^	1–82 547 (18.8%) 2–163 270 (37.2%) 3–103 162 (23.5%) 4–90 001 (20.5%) 5–317 (0.1%)
Mean hours of physical activity per week (SD) (*n* = 439 301)	2.5 (4.6)
Mean years since hypertension diagnosis (SD) (*n* = 103 159)	8.7 (8.2)
Mean SBP in mmHg (SD) (*n* = 439 301)	137.6 (18.6)
Mean automatic SBP in mmHg (SD) (*n* = 425 402)	137.5 (18.6)
Mean manual SBP in mmHg (SD) (*n* = 39 327)	138.2 (18.7)
Mean DBP in mmHg (SD) (*n* = 439 301)	82.2 (10.1)
Mean automatic DBP in mmHg (SD) (*n* = 425 402)	82.1 (10.1)
Mean manual DBP in mmHg (SD) (*n* = 39 327)	82.8 (10.3)

^a^ Education groups coded as follows:

1–None of the above (e.g., primary school completion), 2–O levels/GCSEs or equivalent, 3–CSEs or equivalent, 4–A levels/AS levels or equivalent, 5–NVQ or HND or HNC or equivalent, 6–Other professional qualifications (e.g., teaching, nursing), 7–College or University degree

^b^ Smoking status coded as follows: 1–Never smoked, 2–Previous smoker, 3–Current smoker.

^c^ Hypertension/Antihypertensive use (Hypertension status) coded as follows: 1–No HTN history, no antihypertensive use, 2–No HTN history, currently using antihypertensive, 3–HTN history, currently using antihypertensive, 4–HTN history, no antihypertensive use.

^d^ Current alcohol use coded as follows: 1–Never or on Special occasions only, 2–One to three times a month or once or twice a week, 3–Three or four times a week, 4–Daily or almost daily, 5–Prefer not to answer.

### 3.2 Ideal Blood Pressure and Baseline Cognitive Function

**FIGURE 1:**
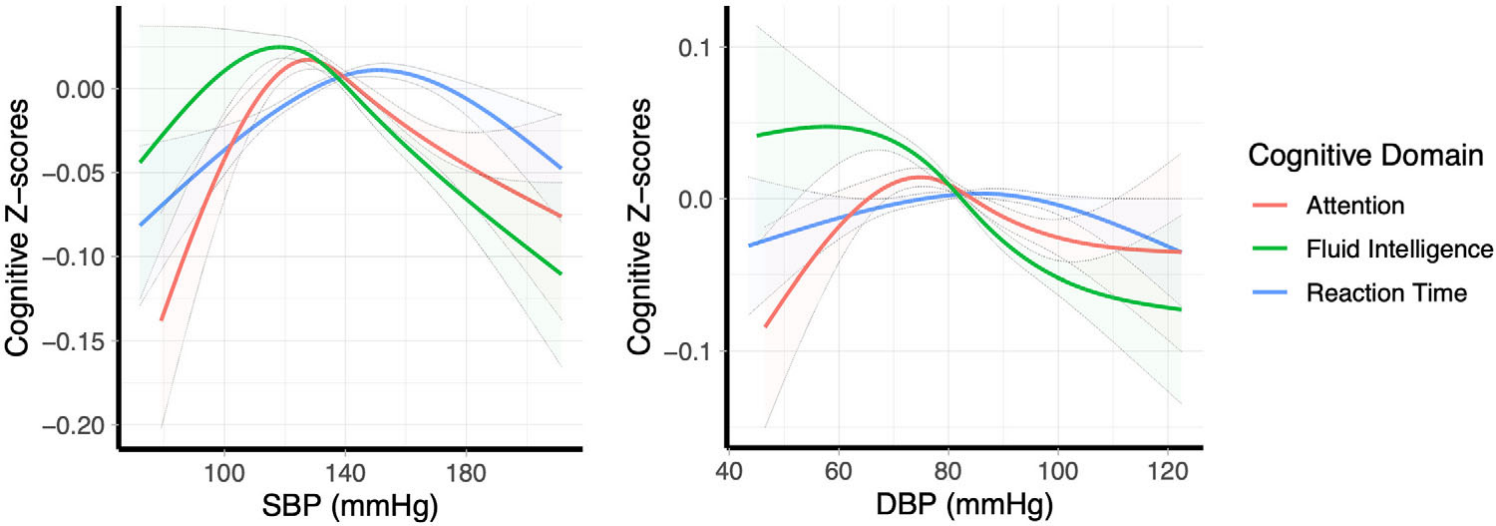
Association between baseline SBP/DBP and the three standardized cognitive outcomes. All natural spline terms included three degrees of freedom. The figure shows the predicted cognitive scores at various points along the range of SBPs and DBPs and the shaded area indicates 95% confidence intervals at those points. For each of the domains, a higher score indicates better performance.

Baseline SBP had a significant association with fluid intelligence (*p* < 0.0001), attention (*p* < 0.0001), and reaction time (*p* < 0.0001) at baseline (Table 2 and Figure 1). Each of these relationships was variation of an inverted U shape with the ideal BP for cognition differing significantly by cognitive domain (fluid intelligence = 118.5 mmHg; 95% CI [78.9–124.0], attention = 127.5 mmHg; 95% CI [124.5–130.5], and reaction time = 150.5 mmHg; 95% CI [145.0–156.0], pairwise‐Wilcoxon test for the difference between ideal BPs, *p* < 0.0001 for all pairs). For each of these, the best nonlinear model included three degrees of freedom (i.e., four knots, a cubic natural spline) and the nonlinear model produced significantly better model fit than the linear model (Table 2, Figure 1 and Figure S1).

**TABLE 2:** Association between baseline SBP/DBP and the three cognitive outcomes.

SBP			
	Predicted fluid intelligence (SD) (95% CI) (*n* =143 979)	Predicted attention (SD) (95% CI) (*n* = 91 404)	Predicted reaction time (SD) (95% CI) (*n* = 427 396)
*p* value	<0.0001^**^	<0.0001^**^	<0.0001^**^
Ideal BP (mmHg)	118.5 (78.9–124)	127.5 (124.5–130.5)	150.5 (145– 156)
**Predicted score at different BP levels**:			
100 mmHg	0.011 (−0.014 to 0.036)	−0.041 (−0.065 to −0.017)	−0.036 (−0.05 to −0.021)
120 mmHg	0.025 (0.018–0.031)	0.012 (0.006–0.018)	−0.01 (−0.013 to −0.006)
140 mmHg	0.002 (−0.002 to 0.006)	0.006 (0.002–0.01)	0.008 (0.006–0.01)
160 mmHg	−0.034 (−0.042 to −0.026)	−0.023 (−0.031 to −0.014)	0.009 (0.004–0.014)
180 mmHg	−0.066 (−0.083 to −0.049)	−0.048 (−0.067 to −0.028)	−0.007 (−0.017 to 0.003)

**DBP**			
	**Predicted fluid score (SD) (95% CI)**	**Predicted attn score (SD) (95% CI)**	**Predicted rt score (SD) (95% CI)**
***p* value**	**<0.0001** ^**^	**<0.0001** ^**^	0.0229
Ideal BP (mmHg)	57.5 (45–69)	74.5 (71–122)	85 (43.5–90.5)
**Predicted score at different BP levels**:			
60 mmHg	0.048 (0.024–0.071)	−0.017 (−0.041 to 0.006)	−0.012 (−0.026 to 0.002)
70 mmHg	0.038 (0.031–0.045)	0.011 (0.004–0.018)	−0.003 (−0.007 to 0.001)
80 mmHg	0.01 (0.005–0.014)	0.01 (0.005–0.014)	0.003 (0–0.005)
90 mmHg	−0.028 (−0.035 to −0.022)	−0.012 (−0.019 to −0.005)	0.003 (−0.001 to 0.006)
100 mmHg	−0.053 (−0.064 to −0.041)	−0.028 (−0.04 to −0.016)	−0.005 (−0.012 to 0.001)

*Note*: The table shows the significance of the overall natural spline term as well as the ideal BP for each cognitive outcome (i.e., the BP at which the cognitive scores are highest). All natural spline terms included three degrees of freedom. The table shows the predicted cognitive scores at various points along the range of SBPs and DBPs as well as the 95% confidence intervals at those points.

* <0.017

** <0.001

Baseline DBP was a significant predictor of fluid intelligence (*p* < 0.0001) and attention (*p* < 0.0001), but not reaction time. The nonlinear model did not produce a significantly better fit than the linear model (*p* = 0.10) when predicting fluid intelligence, suggesting an inverse, approximately linear relationship, with higher BP being associated with lower fluid intelligence (Table S9). The ideal DBP for fluid intelligence (57.5 mmHg; 95% CI [45–69]) and attention (74.5 mmHg; 95% CI [71–122]) also differed significantly, based on pairwise‐Wilcoxon test (*p* < 0.0001) (Table 2, Figure 1, and Figure S2).

### 3.3 Interaction Effect of Age, Sex, and Antihypertensive Use

**FIGURE 2:**
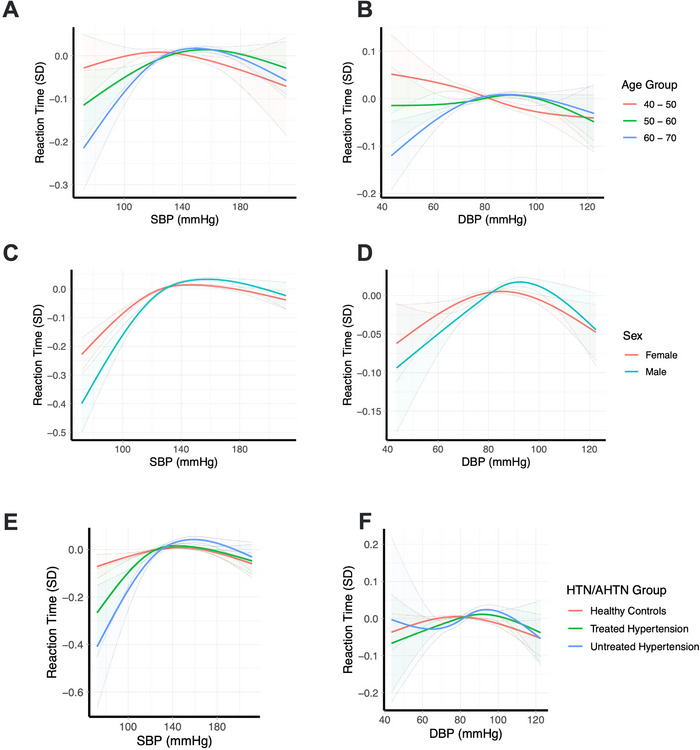
Nonlinear relationship between SBP/DBP and reaction time for significant interactions with age, sex, and hypertension/antihypertensive use groups. A ‐ SBP and Reaction time by age groups; B ‐ DBP and Reaction time by age groups; C ‐ SBP and Reaction time by sex; D ‐ DBP and Reaction time by sex; E ‐ SBP and Reaction time by HTN/AHTN groups; F ‐ DBP and Reaction time by HTN/AHTN groups.

For reaction time, there were significant SBP/DBP interactions with age (*p* < 0.0001), sex (*p* < 0.0001), and hypertension history/antihypertensive use (*p* < 0.0001) (Figure 2 and Tables S10–S12). Those in the 40–50 group showed an inverse, approximately linear relationship between both SBP/DBP and reaction time, whereas individuals in the 50–60 and 60–70 groups increasingly showed worse reaction time at lower SBP and DBP levels (Figure 2A,B). Males with lower BPs (<130/80 mmHg) had significantly worse reaction time compared to females, whereas at BPs > 130/80 mmHg, the relationship was similar for both sexes (Figure 2C,D). In the treated and untreated hypertension groups, individuals with low SBP (i.e., <30 mmHg) had poorer reaction time compared to individuals without hypertension (Figure 2E,F). Conversely, at higher measured SBP, there was little difference between groups based on hypertension status.

**FIGURE 3:**
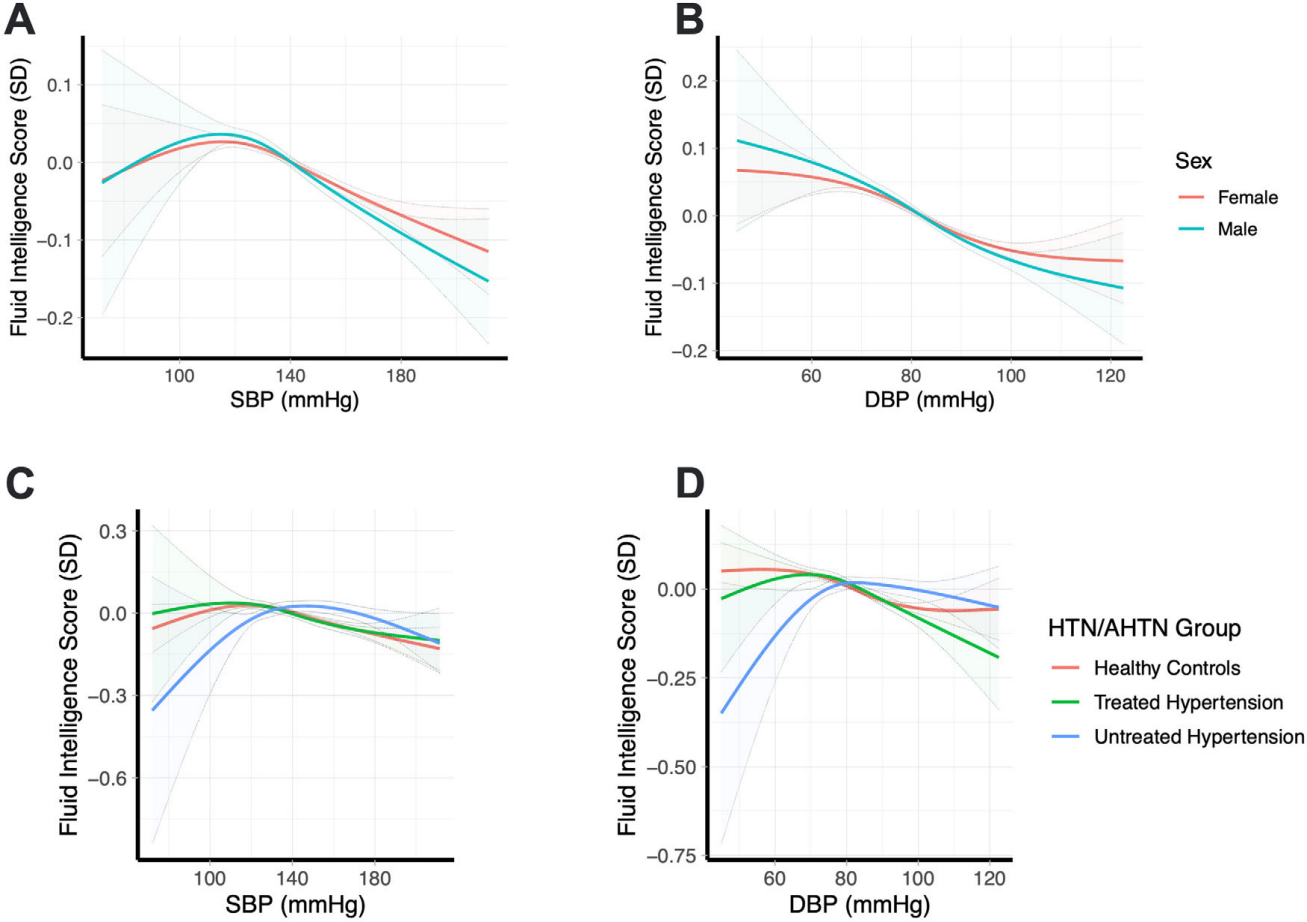
Nonlinear relationship between SBP/DBP and fluid intelligence for significant interactions with sex and hypertension/antihypertensive use groups. A ‐ SBP and Fluid Intelligence by sex; B ‐ DBP and Fluid Intelligence by sex; C ‐ SBP and Fluid Intelligence by HTN/AHTN group; D ‐ DBP and Fluid Intelligence by HTN/AHTN group.

For fluid intelligence, there were significant SBP/DBP interactions with sex and hypertension history/antihypertensive use (Tables S11 and S12). Males had marginally worse fluid intelligence at higher BPs, although the ideal BP and the shapes of the relationships were generally similar for both sexes (Figure 3A,B). Individuals with untreated hypertension and low SBP or DBP (i.e., <130/80 mmHg) had significantly worse fluid intelligence scores compared to individuals without hypertension and those with treated hypertension (Figure 3C,D).

### 3.4 Longitudinal Effects of BP Over Study Course

There was no significant association of baseline SBP or DBP with trajectories of fluid intelligence or reaction time over the course of the study. Higher baseline SBP was associated with a small but significant worsening of attention score trajectory (*p* < 0.0001) over time, with the relationship being approximately inverse linear one (Tables S13 and S14).

### 3.5 Sensitivity Analyses

The sensitivity analyses for the cognitive measures comprising the attention score found that both SBP and DBP were significantly associated with all three of TMTA, TMTB, and DSS with the ideal BPs being 127.5/75 mmHg (95% CI [123/62.9 to 130.5/78.5]), 127/71.5 mmHg (95% CI [121.5/46.5 to 130.5/122.5]), and 129/76.5 mmHg (95% CI [126.5/74.5 to 134.5/122.5]) (Table S15). The shapes of each of the relationships were very similar, corroborating the validity of their grouping into a single cognitive score.

The analysis including PP as the main independent variable was very similar to that of SBP, finding that each of the inverted U‐shaped relationships with fluid intelligence (*p* < 0.0001), attention (*p* < 0.0001), and reaction time (*p* < 0.0001) was significant. Reaction time had a substantially higher ideal PP 64.5 mmHg (95% CI [57.5–69.0]) compared to fluid intelligence 50 mmHg (95% CI [47.0–55.5]) and attention 50.5 mmHg (95% CI [47.5–57.5]) (Tables S16 and S17, Figure S3).

The partially adjusted analysis (Tables S18 and S19, Figure S4) corroborated the main, fully adjusted analysis, but additionally showed a significant nonlinear relationship between DBP and reaction time (*p* < 0.0001). The ideal SBP/DBP was similar to the main analysis for each of fluid intelligence (121.5/65.5 mmHg), attention (128/76.5 mmHg), and reaction time (150/89 mmHg). In the analysis restricted to those with full cognitive outcomes (*n* = 34 635) (Table S20, Figure S5), there was a significant relationship between SBP and all three cognitive outcomes with ideal BPs similar to that of the main, fully adjusted analysis.

## 4 Discussion

Our study found that there were significant nonlinear relationships between BP and fluid intelligence, attention, and reaction time. While each of relationships was approximately an inverted U‐shape, the ideal BP and the rate of decline with changing BP varied considerably.

Fluid intelligence, which was measured by a nontimed 13‐item test of verbal‐numeric reasoning, had an ideal BP of 118.5/57 mmHg, with higher BPs linked to poorer outcomes than lower BPs. By contrast, both outcomes involving timed tests, attention score and reaction time, had significantly higher ideal BPs (127.5/74.5 and 150.5/85 mmHg, respectively) and both high and low BP were associated with similar poorer outcomes. Many previous clinical trials have found that there is a linear relationship between higher BP and cognitive impairment or dementia risk [10, 11]. Similarly, studies have demonstrated an approximately linear relationship between BP and vascular brain pathology, including atherosclerosis, arteriolosclerosis, brain infarcts, lacunes, white matter hyperintensities and tract integrity [12], cerebral microbleeds [13], and blood–brain barrier integrity [14]. Furthermore, a recent secondary analysis of the MRI data from the SPRINT‐MIND clinical trial [15] (mean age [SD] = 67.5 [8.1], *n* = 547) found that intensive BP control (<120 mmHg) resulted in significantly better cerebral perfusion compared to standard control (<140 mmHg).

Given the clinical and pathophysiological link between higher BP and poorer outcomes, why would the current study, among other longitudinal studies of aging [6], find that low, as well as high, BP is deleterious? A common factor underlying both lower BP and poorer cognition is frailty [16], or diminished physiological or homeostatic reserve and impacts BP control and cognitive capacity. Additionally, and linked to frailty, is the possibility of reverse causation, that is, the neurodegeneration associated with dementia causes poorer autonomic control of peripheral BP and thus a declining BP trajectory [17, 18]. However, conversely, there may be a causative relationship between low BP and cognitive decline that is missed within clinicals trials that exclude frail or heavily comorbid participants. Tayler et al. (2023) [19] in a postmortem study of those with various forms of dementia (*n* = 126) argue that higher BPs may help to reduce cerebral ischemia and slow amyloid‐β accumulation in the face of higher cerebrovascular resistance. Supporting this, it has been shown that those who are older and have a history of hypertension have a higher cerebral autoregulatory set point, meaning that the SBP range at which cerebral perfusion is maximized is marginally higher [20, 21]. This is consistent with our sensitivity analyses, which indeed found that those with untreated hypertension had a higher ideal BP for fluid intelligence and reaction time, compared to “healthy controls”.

Although these factors may explain the U‐shape in these relationships, the question remains as to why the ideal BPs were so different, particularly for reaction time. Simple reaction time, unlike other cognitive tests, relies less on cortical, cognitive processing and more on sympathetic activation, peripheral nervous conductivity, and muscle reactivity. In a placebo‐controlled cross‐over study of 12 men [22], Finke and Schächinger found that by activation of the sympathetic nervous system by yohimbine administration significantly increased BP (by 10/8 mmHg) and improved simple reaction time (by 7.5 ms). Conversely, inhibition of the sympathetic nervous system by dexmedetomidine administration decreased BP (by 11/6 mmHg) and worsened simple reaction time (by 7.26 ms). Sympathetic activation causes increases in heart rate, BP, dynamic blood flow, epinephrine release, and skeletal muscle perfusion [23], all of which contribute to faster reaction times. Taken together, these data suggest that rather than higher BPs causing faster reaction time, both are consequence of sympathetic activation [24]. However, it is possible that higher BP itself does increase simple reaction time in a way that has not yet been fully understood or systematically studied in clinical trials. The BP–reaction time relationship is an important target for future research as it has implications for fall risks and other complications from antihypertensives, particularly in elderly, frail patients.

Interestingly, both in this current study and our previous mendelian randomization analysis, we found that males (157.5/92.5 mmHg) had a substantially higher ideal BP for reaction time than females (146.5/85.5 mmHg). This result suggests that physiological differences between males and females may mean that males have better function at higher BPs. Compared to pre‐menopausal females, age‐matched males have twice the prevalence of hypertension [25]. In the long‐term, this increased rate of hypertension contributes to greater cardiovascular and cerebrovascular disease [25], but in the short term it may confer some adaptive reaction time advantage.

This adaptive advantage does seem to be restricted to the shorter term, as other studies have shown that hypertension over longer periods is associated with worsened processing speed [6, 26]. Indeed, in our study, we found that there was a small but significant diminished trajectory of attention score, which in part measured processing speed, over the course of the study in those with higher BPs. These findings are associational and preliminary, involving only very small effect sizes and require further research, including in clinical trial settings, before they may be applied in the management of patients.

### 4.1 Limitations and Strengths

The first limitation of this study is that it relied on a single time‐point measurement of BP, which is highly variable depending on the setting, timing, levels of hydration, stress, and use of manual or automated measurement, among many other factors. This variability reduces the precision with which we can demarcate the relationships. Second, the effects seen in this paper are small to very small, and although the large cohort means that results are significant at a population level, they are not likely to be meaningful at an individual clinical level. Third, as mentioned previously, this is a longitudinal cohort study not an interventional trial and as such the relationships cannot be interpreted as causative. Additionally, the cognitive tests included in this study do not cover all domains included in a general neuropsychological battery and, thus, cognitive function is only partially represented. Within the cognitive domains, the attention score is not pure test of attention, but rather is also a marker of processing speed and executive function and should be interpreted as such. Finally, this study cannot be generalized to non‐White, or non‐European populations as they made up a small fraction of the participant population.

## 5 Conclusion

There is an inverted U‐shaped association between BP and cognitive domains, but the shape of the relationship and the ideal BP differs by cognitive domain. From this and other studies, it is clear that higher BP contributes to cognitive decline, in attention and fluid intelligence. However, cross‐sectionally higher BPs were associated with marginally better reaction times and further research into this BP–reaction time relationship is needed.

## Author Contributions

All authors have contributed to the work, agree with the presented findings, and that the work has not been published before nor is being considered for publication in another journal.

## Ethics Statement

Ethics approval was given by the North West Multi‐Centre Research Ethics Committee (MREC).

## Consent

All human subjects provided informed consent to be involved in the UK Biobank Study.

## Permission to Reproduce Material From Other Sources

No material from other sources was used.

## Conflicts of Interest

The authors declare no conflicts of interest.

## Clinical Trial Registration

No clinical trial registration was required as this was not a clinical trial. Our analysis plan was pre‐registered with Open Science Framework and can be seen here–https://osf.io/8smfz/.

## Supporting information




**Supporting File 1**: jch70129‐sup‐0001‐SuppMat.docx.

## Data Availability

UK Biobank data are available upon application to the UK Biobank here: https://www.ukbiobank.ac.uk/enable‐your‐research/apply‐for‐access. Applications require a specific research plan and access will be given after approval and payment of a fee.
