# Supplementary material for: Does Ideal Blood Pressure Vary by Cognitive Domain? A UK Biobank Study
Source: J Clin Hypertens (Greenwich). 2025 Aug 22;27(8):e70129. doi: 10.1111/jch.70129 (PMC12373150; doi:10.1111/jch.70129)
Supplement: Supplementary file 1 — Supporting File 1: jch70129‐sup‐0001‐SuppMat.docx. [file JCH-27-e70129-s001.docx]

**Supplementary tables**

**Does ideal blood pressure differ by cognitive domain? A UK Biobank Study**

Table of Contents

[Supplementary Methods 3](#_Toc197691965)

[Supplementary table 1: Excluded conditions for which participants were removed 5](#_Toc197691966)

[Supplementary table 2: Descriptions of Cognitive tests 7](#_Toc197691967)

[Supplementary table 3: Results of Principal Components Analysis 8](#_Toc197691968)

[Supplementary table 4: Antihypertensive medications and medication codes taken from UKBB medication history (datafield 20003) 9](#_Toc197691969)

[Supplementary table 5: Numbers and percentages of eligible UKBB participants reporting 12](#_Toc197691970)

[Supplementary table 6: Discrepancy between self-reported touch-screen response to blood pressure medication history and detailed medication history taken by clinician 13](#_Toc197691971)

[Supplementary table 7: Year level equivalents for Educational Qualifications 14](#_Toc197691972)

[Supplementary table 8: Comparing Covariates of the Hypertension Groups 15](#_Toc197691973)

[Supplementary table 9: Individual Natural Splines Terms, Fully-Adjusted Analysis 17](#_Toc197691974)

[Supplementary table 10: Individual Natural Splines Terms (SBP and DBP) including interaction with age, Fully adjusted analysis 18](#_Toc197691975)

[Supplementary table 11: Individual Natural Splines Terms (SBP and DBP) including interaction with sex, Fully adjusted analysis 20](#_Toc197691976)

[Supplementary table 12: Individual Natural Splines Terms (SBP and DBP) including interaction with hypertension history/antihypertensive use, Fully adjusted analysis 21](#_Toc197691977)

[Supplementary table 13: Individual Splines terms SBP/DBP, linear terms and antihypertensive use examining relationships with Cognitive baseline scores and trajectories over time, Fully Adjusted Analysis 23](#_Toc197691978)

[Supplementary table 14: Association between baseline hypertension status/antihypertensive use and the three cognitive outcomes at baseline and over time 25](#_Toc197691979)

[Supplementary table 15: Non-linear relationship between SBP/DBP and TMTA, TMTB and DSS, Fully Adjusted Analysis 26](#_Toc197691980)

[Supplementary table 16: Pulse Pressure and cognitive scores, Fully Adjusted Analysis 27](#_Toc197691981)

[Supplementary table 17: Individual Natural Splines Terms, Pulse Pressure, Fully-Adjusted Analysis 28](#_Toc197691982)

[Supplementary table 18: Non-linear relationship between SBP/DBP and Cognitive outcomes, Partially Adjusted Analysis 29](#_Toc197691983)

[Supplementary table 19: Individual Natural Splines Terms and Linear effect of SBP/DBP on cognitive outcomes, Partially Adjusted Analysis 30](#_Toc197691984)

[Supplementary table 20: Non-linear relationship between SBP/DBP and Cognitive outcomes, restricted analysis in those with complete cognitive scores 31](#_Toc197691985)

[Supplementary Figure 1: Non-linear relationship between SBP and Cognitive Outcomes, Fully Adjusted Analysis 32](#_Toc197691986)

[Supplementary Figure 2: Non-linear relationship between DBP and Cognitive Outcomes, Fully Adjusted Analysis 33](#_Toc197691987)

[Supplementary Figure 3: Non-linear relationship between PP and Cognitive Outcomes, Fully Adjusted Analysis 34](#_Toc197691988)

[Supplementary Figure 4: Non-linear relationship between SBP/DBP and Cognitive Outcomes, Partially Adjusted Analysis 35](#_Toc197691989)

[Supplementary Figure 5: Non-linear relationship between SBP/DBP and Cognitive Outcomes, Analysis restricted to those with complete outcomes 36](#_Toc197691990)

## Supplementary Methods

*Hypertension/Antihypertensive use Groups*

There were a number of reasons the the “uncertain hypertension” group was considered anomalous. First, they may have been unaware of a previous diagnosis of hypertension despite taking antihypertensive treatment. Second, they may have been taking the antihypertensive for a reason other than hypertension (e.g. kidney disease, arrhythmias, palpitations, heart failure). Given that their previous hypertension history status was unclear they were excluded from analyses that included this variable.

*Assessment of Antihypertensive Use*

Antihypertensive use was assessed in two ways. Participants were asked on a touchscreen "Do you regularly take any of the following medications? (You can select more than one answer)" with one of the responses being “blood pressure medication) (UKBB Data fields [6177](https://biobank.ndph.ox.ac.uk/ukb/field.cgi?id=6177) (n = 227363) and 6153 (n = 271,298) (n total = 498,661). In a smaller group of participants, a comprehensive medication history (UKBB Data field 20003) was taken by a clinician (n = 380,136) with each type of medication itemised. The list of medications included or defined as antihypertensives and their respective codes are included in Supplementary table 4 and the numbers of participants taking each of the subtypes of medications are outlined in Supplementary table 5. There was some discrepancy between those who reported either taking or not taking blood pressure medication and their itemised medication list, with overall concordance of the two measures being 93.8% (Supplementary table 7). Because the more detailed, itemised medication history taken by a clinician was thought to be more reliable, if medication history data was available, it was utilised in preference to the single touch screen question.

*Coding of covariates*

Age was grand-mean centred (at 56) and, to better model non-linear changes of cognition with age, a quadratic age term was included in all analyses. Sex was coded as a binary variable (0–Female, 1–Male). The Townsend deprivation index is a measure of material deprivation based on the participant’s living location. It was included in all models as a continuous variable. Educational attainment was initially coded as a 7-level categorical variable (College or University degree, A levels/AS levels or equivalent, O levels/GCSEs or equivalent, CSEs or equivalent, NVQ or HND or HNC or equivalent, Other professional qualifications, e.g., nursing, teaching, None of the above). These were transformed into a continuous measure of years of education (Supplementary Table 7). The assessment centre was included as a random effect, categorical variable (22 levels). History of cardiovascular disease was included as a dummy variable (0-no, 1-yes). Those who self-reported a history of diabetes or reported being on an anti-diabetic medication were defined as having “diabetes” and this was included as a dummy variable (0-no, 1-yes). A self-reported history of high cholesterol, as indicated by whether the individual takes cholesterol medication, was included as a dummy variable (0-does not take medication for high cholesterol, 1-does take medication for high cholesterol).

Current physical activity was self-reported at baseline and was included in models as a continuous variable indicating the average number of hours per week in which an individual was moderately or vigorously exercising. Alcohol use was measured by self-reported frequency of use and was coded as a 5-level categorical variable (1–Never or on Special occasions only, 2-One to three times a month or Once or twice a week, 3-Three or four times a week, 4-Daily or almost daily, 5-Prefer not to answer). Smoking was defined as a 3-level categorical variable (1-never smoker, 2-previous smoking, 3-current smoker). Body Mass Index (Weight in kg/(Height in m squared)) was treated as a continuous variable.

*Cognitive Test Data*

All tests were examined visually for Gaussian distribution and if skewness was >1 or <-1 the test was transformed. All tests were winsorised to within 4 standard deviations of the mean to control for outliers and then z-transformed to provide a standardised measure.

Multiple imputation for missing data was considered but given that for many of the participants it is the outcome that is missing and the numbers of participants completing cognitive tests were so different it was unfeasible.

| **Wave** | **Mean years in Study (SD)** | **Fluid intelligence** | **Reaction time** | **Digit Symbol Substitution** | **TMTA** | **TMTB** |
| --- | --- | --- | --- | --- | --- | --- |
| 1 | 0 (0) | 158570 | 476453 | 113572 | 99785 | 99783 |
| 2 | 4.31 (0.92) | 19276 | 19421 | 0 | 0 | 0 |
| 3 | 8.98 (1.76) | 43198 | 43792 | 31700 | 32041 | 32041 |
| 4 | 11.06 (1.05) | 3460 | 3473 | 3468 | 3554 | 3554 |

*Interpretation of Natural Splines Terms*

Natural splines utilise a combination of polynomial functions that are joined together smoothly at knots by ensuring continuity of first and second differentials at these points. Before the first knot and after the last knot, the spline is constrained to be close to linear to avoid overestimation of effects at the extremes of the population. The additional linearity ensures that the shape of the curve is less erratic and more reliable. Natural splines with degrees of freedom varying between 2 and 4 (i.e. between 3 and 5 knots) were used and the model with best fit, based on Akaike Information Criteria (AIC), was selected.

The piecewise construction of the continuous model is defined by the truncated power function which set out below:


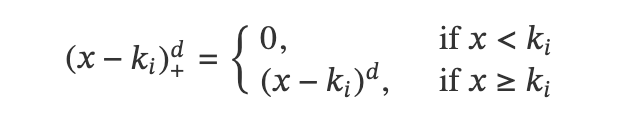


Applying this above power function, a cubic spline regression with 4 knots (two internal knots and two boundary knots) can be defined with the following formula. Each of the three segments will have a different set of coefficients for each segment of the curve.

𝑦 = 𝛽0 + 𝛽1𝑥 + 𝛽2𝑥^2^ +𝛽3𝑥^3^ + 𝛽4(𝑥−𝑘1)^3^ + 𝛽5(𝑥−𝑘2)^3^ + 𝛽6(𝑥−𝑘3)^3^ + 𝛽7(𝑥−𝑘4)^3^ + 𝜀

In the tables included within this supplement the terms NS(SBP, 3)1, NS(SBP, 3)2, and NS(SBP, 3)3 correspond to the weights of the three separate basis functions that define each section of the curve. Given the complexity of the formula, interpretation of the individual coefficients and their p-values is challenging and thus it is generally better to assess the natural splines relationship in a graph, as we have done.

## Supplementary table 1: Excluded conditions for which participants were removed

| **Excluded illnesses** |
| --- |
| Brain cancer/primary malignant tumour  Brain haemorrhage  Brain/intracranial abscess  Cerebral aneurysm  Cerebral palsy  Chronic/degenerative neurological problem  Dementia/Alzheimer's disease/cognitive impairment  Encephalitis  Epilepsy  Infection of nervous system  Ischaemic stroke  Meningeal cancer/malignant meningioma  Meningioma (benign)  Meningitis  Motor neurone disease  Multiple sclerosis  Neurological injury/trauma  Neuroma (benign)  Other demyelinating condition  Other neurological problem  Parkinson's disease  Spina bifida  Stroke  Subarachnoid haemorrhage  Subdural haematoma |

## Supplementary table 2: Descriptions of Cognitive tests

| **Fluid intelligence (n = 165,447)-** A task with 13 logic/reasoning-type questions and a 2-min time limit. The maximum score is 13. Participants who did not answer all of the questions within the allotted 2-minute limit are scored as zero for each of the unattempted questions. |
| --- |
| **Trail making test A and B (n = 104,029)–** Time was measured to complete the task. Higher times indicate poorer outcomes. There are also measures of how many errors occurred but this is a less well validated tool and errors contribute to longer times to completion anyhow and are incorporated this way. (These are part of the online follow up). |
| **Digit Symbol Substitution Test (n = 118,450)** – the number of correctly associated digits with symbols in the online version. The number attempted is given by ID number (20195) for which the number correct should be moderated with. |
| **Reaction Time (n = 496,768)** - participants completed a timed test of symbol matching, similar to the common card game ‘Snap’. This field is the mean duration to first press of snap-button summed over rounds in which both cards matched. It gives a crude measure of the raw processing and reaction speed of a participant.  The following data points were excluded when forming the average:   - Rounds 0-4 were regarded as "training"; - Times under 50ms must be due to anticipation rather than reaction; - Times over 2000ms were ignored as the cards had disappeared by then.   Values were rounded to the nearest whole number. |

## Supplementary table 3: Results of Principal Components Analysis

|  | **Dim.1** | **Dim.2** | **Dim.3** | **Dim.4** | **Dim.5** | **Dim.6** |
| --- | --- | --- | --- | --- | --- | --- |
| **Reaction Time** | 0.1323 | 0.0114 | 0.6849 | 0.0109 | 0.0113 | 0.0001 |
| **Fluid Intelligence** | 0.3722 | 0.4147 | 0.0008 | 0.2417 | 0.0182 | 0.0033 |
| **Digit Symbol Substitution** | 0.5168 | 0.0539 | 0.0002 | 0.0138 | 0.3465 | 0.007 |
| **Trail Making Test A** | 0.522 | 0.1749 | 0.0486 | 0.0068 | 0.0978 | 0.1194 |
| **Trail Making Test B** | 0.678 | 0.0434 | 0.0251 | 0.002 | 0.0193 | 0.2042 |

**Supplementary table 3:** Results of rotated principle components analysis showing that DSS, TMTA and TTMB loaded onto the first factor and both fluid intelligence and reaction time were sufficiently independent constructs to load onto their own factor.

## Supplementary table 4: Antihypertensive medications and medication codes taken from UKBB medication history (datafield 20003)

| **UKBB Code** | **Medication** |
| --- | --- |
| **Angiotensin Converting Enzyme Inhibitor** | |
| 1140860806 | Ramipril |
| 1141165470 | felodipine+ramipril |
| 1140860696 | lisinopril |
| 1140864952 | lisinopril+hydrochlorothiazide 10mg/12.5mg tablet |
| 1140888560 | perindopril |
| 1141180592 | perindopril+indapamide |
| 1140860802 | coversyl 2mg tablet perindopril erbumine |
| 1140888552 | enalapril |
| 1140860790 | enalapril maleate+hydrochlorothiazide 20mg/12.5mg tablet |
| 140860904 | trandolapril |
| 1141153328 | trandolapril+verapamil hydrochloride |
| 1140888556 | fosinopril |
| 1140860750 | captopril |
| 1140860764 | captopril+hydrochlorothiazide 25mg/12.5mg tablet |
| 1140860728 | quinapril |
| 1141164154 | tanatril 5mg tablet) (imidapril) |
| **Angiotensin Receptor Blocker** | |
| 1141156836 | candesartan cilexetil |
| 1140916356 | losartan |
| 1141152998 | irbesartan |
| 1141145660 | valsartan |
| 1141166006 | telmisartan |
| 1141193282 | olmesartan |
| 1141145668 | (valsartan) |
| 1141153006 | aprovel 75mg tablet (irbesartan) |
| 1141151016 | losartan potassium+hydrochlorothiazide 50mg/12.5mg tablet |
| 1141193346 | olmetec 10mg tablet (olmesartan) |
| 1141171336 | eprosartan |
| 1141172682 | irbesartan+hydrochlorothiazide 150mg/12.5mg tablet |
| 1141201038 | valsartan+hydrochlorothiazide 80mg/12.5mg tablet |
| 1141187788 | telmisartan+hydrochlorothiazide 40mg/12.5mg tablet |
| **Calcium Channel Blocker** | |
| 1140879802 | amlodipine |
| 1140888646 | felodipine |
| 1140861088 | nifedipine |
| 1140879806 | diltazem |
| 1140879818 | metoprolol |
| 1140888510 | verapimil |
| 1140861138 | (diltiazem) |
| 1140909368 | carvedilol |
| 1141153032 | zanidip 10mg tablet (lercandipine) |
| 1140861202 | istin 5mg tablet |
| 1140923572 | adipine mr 10 m/r tablet (nifedipine) |
| 1140911698 | slozem 120mg m/r capsule (diltiazem) |
| 1140917428 | angitil sr 90 m/r capsule (diltiazem) |
| 1140926778 | diltiazem hcl+hydrochlorothiazide 150mg/12.5mg m/r capsule |
| 1140879810 | nicardipine |
| 1141165470 | felodipine+ramipril |
| 1141157140 | nifedipress mr 10 m/r tablet |
| 1141153328 | trandolapril+verapamil hydrochloride |
| 1141150926 | verapress mr 240 m/r tablet (verapamil) |
| 1140860426 | atenolol+nifedipine 50mg/20mg m/r capsule |
| 1141187774 | vera til sr 120mg m/r tablet (verapamil) |
| 1141169730 | nifopress retard 20mg m/r tablet |
| **Diuretic** | |
| 1140909708 | furosemide |
| 1140866116 | frusemide |
| 1140866236 | spironolactone |
| 1140866078 | indapamide |
| 1141180592 | perindopril+indapamide |
| 1141151016 | losartan potassium+hydrochlorothiazide 50mg/12.5mg tablet |
| 1140864952 | lisinopril+hydrochlorothiazide 10mg/12.5mg tablet |
| 1140860790 | enalapril maleate+hydrochlorothiazide 20mg/12.5mg tablet |
| 1141201038 | valsartan+hydrochlorothiazide 80mg/12.5mg tablet |
| 1140926778 | diltiazem hcl+hydrochlorothiazide 150mg/12.5mg m/r capsule |
| 1141187788 | telmisartan+hydrochlorothiazide 40mg/12.5mg tablet |
| 1140860332 | sotalol hydrochloride+hydrochlorothiazide 80mg/12.5mg tablet |
| 1140860764 | captopril+hydrochlorothiazide 25mg/12.5mg tablet |
| **Beta-Blockers** | |
| 1140866738 | atenolol |
| 1140879760 | bisoprolol |
| 1140879760 | bisoprolol |
| 1140879842 | propranolol |
| 140864950 | bisoprolol fumarate+hydrochlorothiazide 10mg/6.25mg tablet |
| 1141164276 | nebivolol |
| 1140879762 | celiprolol |
| 1140879824 | labetalol |
| 1140860332 | sotalol hydrochloride+hydrochlorothiazide 80mg/12.5mg tablet |
| 1141146126 | atenolol+bendrofluazide |
| 1140860308 | metoprolol tartrate+chlorthalidone 100mg/12.5mg tablet |
| 1140860192 | nadolol |
| 1140860426 | atenolol+nifedipine 50mg/20mg m/r capsule |
| 1140860492 | emcor 10mg tablet (bisprolol) |
| 1140860274 | lopresor 50mg tablet (metoprolol) |
| 1141146128 | atenolol+co amilozide |
| 1141146124 | atenolol+chlorthalidone |
| 1140875808 | metipranolol |
| **Alpha 1 Agonist/Alpha 2 Antagonist** | |
| 1140883468 | clonidine |
| 1140879798 | terazosin |
| 1140879794 | prazosin |
| **Other** | |
| 1140888762 | ismn isosorbide mononitrate |
| 1140860976 | imdur 60mg durule |
| 1140910512 | ismo isosorbide mononitrate |
| 1141172698 | monomax xl 60mg m/r tablet (isosorbide mononitrate) |
| 1140861008 | isosorbide dinitrate |
| 1141157254 | isosorbide mononitrate product |
| 1140923788 | monomax sr 40 m/r capsule |
| 1140888686 | hydralazine |
| 1141162550 | monosorb xl 60 m/r tablet |
| 1141192364 | carmil xl 60mg m/r tablet (isosorbide mononitrate) |
| 1140861024 | sorbitrate 10mg tablet |
| 1140910606 | alpha methyldopa |

**Supplementary table 4:** Medication codes and medications included as antihypertensives from the UK biobank.

## Supplementary table 5: Numbers and percentages of eligible UKBB participants reporting

| **Medication Class** | **N (%) of participants taking the medication** |
| --- | --- |
| Angiotensin II Converting Enzyme Inhibitors | 41131 (13.1%) |
| Angiotensin Receptor Blockers | 16315 (5.2%) |
| Calcium Channel Blockers | 25731 (8.2%) |
| Beta-Blockers | 25208 (8%) |
| Alpha 1 Blockers | 716 (0.2%) |
| Other | 832 (0.3%) |
| Frusemide | 3521 (1.1%) |
| Spironolactone | 710 (0.2%) |
| Thiazides | 2186 (0.7%) |

## Supplementary table 6: Discrepancy between self-reported touch-screen response to blood pressure medication history and detailed medication history taken by clinician

|  | **Touch-screen self-report - not taking blood pressure medication** | **Touch-screen self-report - taking blood pressure medication** |
| --- | --- | --- |
| **Medication history - not taking blood pressure medication** | 223198 (69.6%) | 12595 (3.9%) |
| **Medication history - Taking blood pressure medication** | 8378 (2.6%) | 76652 (23.9%) |
| **Overall Concordance** | 93.8% | |

**Supplementary table 6:** Portion of participants reporting taking anti-hypertensive medication from two different questions within the UK Biobank study. Antihypertensive use was assessed in two ways. Participants were asked on a touchscreen "Do you regularly take any of the following medications? (You can select more than one answer)" with one of the responses being “blood pressure medication) (UKBB Data fields 6177 (n = 227363) and 6153 (n = 271,298) (n total = 498,661). In a smaller group of participants, a comprehensive medication history (UKBB Data field 20003) was taken by a clinician (n = 380,136) with each type of medication itemised. The list of medications included or defined as antihypertensives and their respective codes are included in Supplementary table 5. The above table details the discrepancy between the responses from those two measures.

## Supplementary table 7: Year level equivalents for Educational Qualifications

| **Educational Attainment** | **Years of Education (minimum)** |
| --- | --- |
| O levels/GCSEs or equivalent | 10 |
| CSEs or equivalent | 10 |
| A levels/AS levels or equivalent | 12 |
| NVQ or HND or HNC or equivalent | 13 |
| Other professional qualifications (e.g. teaching, nursing) | 14 |
| College or University degree | 16 |
| None of the above (i.e. primary school completed) | 6 |

**Supplementary table 7:** Equivalents of British qualifications in years of education. Note in this it is assumed that everyone in the UK was at least schooled to a primary school level.

## Supplementary table 8: Comparing Covariates of the Hypertension Groups

|  | **Total** | **"Healthy Controls"** | **"Uncertain Hypertension"** | **"Treated Hypertension"** | **"Untreated Hypertension"** | **P-value** |
| --- | --- | --- | --- | --- | --- | --- |
|  | **n=439,301** | **n=321844** | **n=7402** | **n=82281** | **n=35673** |  |
| **Mean Age (SD) (n=439,301)** | 56.3 (8.1) | 55.1 (8.1) | 61.2 (6.5) | 60.5 (6.4) | 56.3 (7.7) | **<0.0001**** |
| **Sex (M%) (n=439,301)** | 45.10% | 42.40% | 69.80% | 51.80% | 48.80% | **<0.0001**** |
| **Mean Days in Study (SD) (n=439,301)** | 376.7 (1049.5) | 406.5 (1086.6) | 253.7 (861.5) | 270.3 (886.7) | 342.1 (1002.4) | **<0.0001**** |
| **Education Group (n=439,301)^a^** | 1 - 73963 (16.7%)  2 - 50455 (11.4%)  3 - 24764 (5.6%)  4 - 24631 (5.6%)  5 - 56972 (12.8%)  6 - 65455 (14.8%)  7 - 147351 (33.2%) | 1 - 44647 (14%)  2 - 35439 (11.1%)  3 - 18906 (5.9%)  4 - 18235 (5.7%)  5 - 40886 (12.8%)  6 - 46634 (14.6%)  7 - 114200 (35.8%) | 1 - 2101 (28.9%)  2 - 793 (10.9%)  3 - 255 (3.5%)  4 - 349 (4.8%)  5 - 933 (12.8%)  6 - 1080 (14.9%)  7 - 1755 (24.2%) | 1 - 20582 (25.4%)  2 - 10053 (12.4%)  3 - 3383 (4.2%)  4 - 3991 (4.9%)  5 - 10141 (12.5%)  6 - 12521 (15.4%)  7 - 20470 (25.2%) | 1 - 6308 (17.9%)  2 - 4058 (11.5%)  3 - 2168 (6.1%)  4 - 2013 (5.7%)  5 - 4903 (13.9%)  6 - 5116 (14.5%)  7 - 10697 (30.3%) | **<0.0001**** |
| **Mean Townsend Deprivation Index (SD) (n=439,301)** | -1.3 (3.1) | -1.4 (3) | -1 (3.2) | -1.1 (3.2) | -1.1 (3.2) | **<0.0001**** |
| **Diabetes History (%) (n=439,298)** | 5.70% | 2.70% | 20.90% | 16.10% | 5.20% | **<0.0001**** |
| **Smoking History (n and %) (n=437,820)^b^** | 1 - 248386 (55.2%)  2 - 154743 (34.4%)  3 - 46744 (10.4%) | 1 - 182948 (57%)  2 - 103450 (32.2%)  3 - 34408 (10.7%) | 1 - 3149 (42.9%)  2 - 3396 (46.2%)  3 - 801 (10.9%) | 1 - 41233 (50.4%)  2 - 33606 (41%)  3 - 7034 (8.6%) | 1 - 18730 (52.7%) 2 - 12950 (36.4%) 3 - 3874 (10.9%) | **<0.0001**** |
| **CVD History (%) (n=438,454)** | 4.20% | 1.60% | 50.10% | 11.10% | 2.50% | **<0.0001**** |
| **Mean BMI (%) (n=437,796)** | 27.4 (4.8) | 26.6 (4.4) | 28.8 (4.9) | 29.8 (5.3) | 28.6 (5) | **<0.0001**** |
| **Current Alcohol Use (n=439,297)^c^** | 1 - 86720 (19.2%)  2 - 167579 (37.1%)  3 - 105192 (23.3%)  4 - 91663 (20.3%)  5 - 500 (0.1%) | 1 - 57942 (18%)  2 - 122295 (38%)  3 - 77220 (24%)  4 - 64164 (19.9%)  5 - 223 (0.1%) | 1 - 1817 (24.5%)  2 - 2582 (34.9%)  3 - 1534 (20.7%)  4 - 1463 (19.8%)  5 - 6 (0.1%) | 1 - 19116 (23.2%)  2 - 28314 (34.4%)  3 - 17252 (21%)  4 - 17510 (21.3%)  5 - 89 (0.1%) | 1 - 6773 (19%)  2 - 12834 (36%)  3 - 8299 (23.3%)  4 - 7726 (21.7%)  5 - 41 (0.1%) | **<0.0001**** |
| **Hours of Physical Activity per week (n=439,301)** | 2.5 (4.6) | 2.7 (4.7) | 2.2 (4.5) | 2 (4.2) | 2.6 (4.8) | **<0.0001**** |
| **Mean years since hypertension diagnosis (SD) (n=103,159)** | 8.7 (8.2) | - | - | 9.4 (8) | 7.1 (8.4) | **<0.0001**** |
| **Mean SBP in mmHg (SD) (n=439,301)** | 137.6 (18.6) | 134.2 (17.5) | 135.2 (18.1) | 145.9 (17.6) | 150.2 (18.7) | **<0.0001**** |
| **Mean DBP in mmHg (SD) (n=439,301)** | 82.2 (10.1) | 80.8 (9.7) | 78.3 (10.1) | 85 (9.8) | 89.7 (10.1) | **<0.0001**** |

**Supplementary table 8:** Comparison of the four hypertension history/antihypertensive use groups. Because the uncertain hypertension group were excluded from the analysis the p-values on the side represent an assessment of the significance of the difference between “Healthy Controls”, Treated Hypertension and Untreated Hypertension. For continuous variables comparison was made by 2-tailed ANOVA and for categorical variables chi-squared analysis was used.

^a^Education groups coded as follows

1 - None of the above (e.g. primary school completion), 2 - O levels/GCSEs or equivalent, 3 - CSEs or equivalent, 4 - A levels/AS levels or equivalent, 5 - NVQ or HND or HNC or equivalent, 6 - Other professional qualifications (e.g. teaching, nursing), 7 - College or University degree

^b^Smoking status coded as follows: 1 - Never smoked, 2 – Previous smoker, 3 - Current smoker).

^c^Current Alcohol use coded as follows: (1 – Never or on Special occasions only, 2 - One to three times a month or Once or twice a week, 3 - Three or four times a week, 4 - Daily or almost daily, 5 - Prefer not to answer).

## Supplementary table 9: Individual Natural Splines Terms, Fully-Adjusted Analysis

|  | **Fluid Intelligence** | | **Attention** | | **Reaction Time** | |
| --- | --- | --- | --- | --- | --- | --- |
|  | **B (95%CI)** | **P value** | **B (95%CI)** | **P value** | **B (95%CI)** | **P value** |
| **SBP** | | | | | | |
| **p-value v null** |  | **<0.0001**** |  | **<0.0001**** |  | **<0.0001**** |
| **p-value v linear** |  | **0.0022*** |  | **<0.0001**** |  | **<0.0001**** |
| **NS(SBP, 3)1** | -0.036 (-0.076, 0.005) | 0.0857 | 0.055 (0.016, 0.095) | 0.0062 | 0.077 (0.054, 0.101) | **<0.0001**** |
| **NS(SBP, 3)2** | 0.047 (-0.127, 0.221) | 0.5956 | 0.307 (0.135, 0.48) | **0.0005**** | 0.125 (0.024, 0.227) | **0.0154*** |
| **NS(SBP, 3)3** | -0.115 (-0.187, -0.044) | **0.0016*** | -0.018 (-0.095, 0.058) | 0.6376 | -0.005 (-0.047, 0.037) | 0.815 |
| **DBP** | | | | | | |
| **p-value v null** |  | **<0.0001**** |  | **<0.0001**** |  | 0.0229 |
| **p-value v linear** |  | 0.0959 |  | **0.0007**** |  | **0.0085*** |
| **NS(DBP, 3)1** | -0.108 (-0.146, -0.069) | **<0.0001**** | 0.013 (-0.025, 0.05) | 0.5142 | 0.025 (0.003, 0.048) | 0.027 |
| **NS(DBP, 3)2** | -0.093 (-0.263, 0.076) | 0.2817 | 0.187 (0.018, 0.356) | 0.0303 | 0.036 (-0.063, 0.135) | 0.4767 |
| **NS(DBP, 3)3** | -0.126 (-0.202, -0.049) | **0.0013*** | -0.014 (-0.093, 0.065) | 0.7283 | -0.025 (-0.069, 0.019) | 0.2574 |
|  | **TMTA** | | **TMTB** | | **DSS** | |
| **SBP** | | | | | | |
| **p-value v null** |  | **<0.0001**** |  | **<0.0001**** |  | **<0.0001**** |
| **p-value v linear** |  | **<0.0001**** |  | **<0.0001**** |  | **<0.0001**** |
| **NS(SBP, 3)1** | 0.056 (0.006, 0.106) | 0.028 | 0.038 (-0.009, 0.086) | 0.1147 | 0.075 (0.031, 0.119) | **0.0009**** |
| **NS(SBP, 3)2** | 0.328 (0.111, 0.546) | **0.0031*** | 0.235 (0.026, 0.444) | 0.0275 | 0.349 (0.156, 0.541) | **0.0004**** |
| **NS(SBP, 3)3** | -0.031 (-0.128, 0.066) | 0.5324 | -0.048 (-0.141, 0.045) | 0.3074 | 0.009 (-0.076, 0.094) | 0.8281 |
| **DBP** | | | | | | |
| **p-value v null** |  | **0.0001**** |  | **0.0001**** |  | **0.0005**** |
| **p-value v linear** |  | **0.0043*** |  | 0.1015 |  | **0.0003**** |
| **NS(DBP, 3)1** | 0.016 (-0.032, 0.064) | 0.5118 | -0.023 (-0.068, 0.023) | 0.3358 | 0.053 (0.011, 0.095) | **0.0126*** |
| **NS(DBP, 3)2** | 0.174 (-0.039, 0.388) | 0.11 | 0.085 (-0.12, 0.29) | 0.415 | 0.31 (0.123, 0.498) | **0.0012*** |
| **NS(DBP, 3)3** | -0.039 (-0.14, 0.061) | 0.4414 | -0.036 (-0.132, 0.061) | 0.4676 | 0.036 (-0.052, 0.125) | 0.4231 |

**Supplementary table 9:** Individual SBP/DBP natural splines terms for the three main cognitive outcomes (Fluid Intelligence, Attention and Reaction time) as well as the three cognitive outcomes making up the attention score. The table shows the significance of the overall natural splines term (p-value v null) and whether the natural splines term produced a significantly better model fit compare to a linear term for BP (p-value v linear). All natural splines terms included three degrees of freedom. Analyses were adjusted for age, age^2^, sex, the Townsend deprivation index, education, cardiovascular disease history, diabetes status, smoking status, current physical activity, current alcohol use frequency, BMI and history of hypertension/antihypertensive use.

TMTA - Trail Making Test A; TMTB – Trail Making Test B; DSS – Digit Symbol Substitution test.

*<0.017

**<0.001

## Supplementary table 10: Individual Natural Splines Terms (SBP and DBP) including interaction with age, Fully adjusted analysis

|  | **Fluid Intelligence (n = 143,979)** | | **Attention (n = 91,404)** | | **Reaction Time (n = 427,396)** | |
| --- | --- | --- | --- | --- | --- | --- |
| **SBP** | | | | | | |
|  | **B (95%CI)** | **P value** | **B (95%CI)** | **P value** | **B (95%CI)** | **P value** |
| **Age 40-50** | - | - | - | - | - | - |
| **Age 50-60** | 0.222 (0.03, 0.414) | 0.0236 | -0.245 (-0.43, -0.061) | **0.009*** | -0.425 (-0.536, -0.314) | **<0.0001**** |
| **Age 60-70** | 0.029 (-0.184, 0.241) | 0.7921 | -0.888 (-1.105, -0.67) | **<0.0001**** | -0.884 (-1.008, -0.76) | **<0.0001**** |
| **NS(SBP, 3)1** | -0.007 (-0.071, 0.056) | 0.8253 | 0.084 (0.02, 0.147) | 0.0098 | 0.001 (-0.036, 0.038) | 0.9484 |
| **NS(SBP, 3)2** | 0.099 (-0.221, 0.418) | 0.5455 | 0.495 (0.164, 0.825) | **0.0033*** | 0.026 (-0.158, 0.21) | 0.781 |
| **NS(SBP, 3)3** | -0.162 (-0.386, 0.063) | 0.1577 | 0.086 (-0.165, 0.337) | 0.5018 | -0.065 (-0.192, 0.061) | 0.3101 |
| **Age Interaction – P-value** |  | 0.5682 |  | 0.6101 |  | **<0.0001**** |
| **NS(SBP, 3)1* Age 50-60** | -0.058 (-0.149, 0.032) | 0.2063 | -0.067 (-0.153, 0.019) | 0.1262 | 0.101 (0.049, 0.154) | **0.0001**** |
| **NS(SBP, 3)2* Age 50-60** | -0.251 (-0.69, 0.189) | 0.2643 | -0.352 (-0.786, 0.083) | 0.1125 | 0.177 (-0.077, 0.43) | 0.1715 |
| **NS(SBP, 3)3* Age 50-60** | 0 (-0.262, 0.262) | 0.9992 | -0.174 (-0.459, 0.111) | 0.2319 | 0.101 (-0.047, 0.249) | 0.1813 |
| **NS(SBP, 3)1* Age 60-70** | -0.005 (-0.108, 0.099) | 0.9258 | 0.006 (-0.099, 0.112) | 0.9043 | 0.162 (0.102, 0.223) | **<0.0001**** |
| **NS(SBP, 3)2* Age 60-70** | 0.105 (-0.362, 0.572) | 0.6589 | -0.068 (-0.555, 0.418) | 0.783 | 0.359 (0.087, 0.631) | **0.0096*** |
| **NS(SBP, 3)3* Age 60-70** | 0.082 (-0.167, 0.332) | 0.518 | -0.077 (-0.355, 0.202) | 0.5882 | 0.118 (-0.024, 0.26) | 0.1024 |
| **DBP** | | | | | | |
|  | **B (95%CI)** | **P value** | **B (95%CI)** | **P value** | **B (95%CI)** | **P value** |
| **Age 40-50** | - | - | - | - | - | - |
| **Age 50-60** | 0.175 (-0.027, 0.377) | 0.0888 | -0.338 (-0.528, -0.148) | **0.0005**** | -0.407 (-0.524, -0.291) | **<0.0001**** |
| **Age 60-70** | -0.129 (-0.318, 0.06) | 0.1822 | -0.878 (-1.07, -0.686) | **<0.0001**** | -0.868 (-0.978, -0.758) | **<0.0001**** |
| **NS(DBP, 3)1** | -0.135 (-0.205, -0.066) | **0.0001**** | 0.041 (-0.026, 0.108) | 0.2305 | -0.072 (-0.112, -0.032) | **0.0004**** |
| **NS(DBP, 3)2** | -0.173 (-0.494, 0.149) | 0.2929 | 0.238 (-0.076, 0.552) | 0.1376 | -0.108 (-0.291, 0.074) | 0.2438 |
| **NS(DBP, 3)3** | -0.164 (-0.318, -0.01) | 0.0365 | -0.017 (-0.178, 0.145) | 0.8408 | -0.081 (-0.167, 0.005) | 0.0635 |
| **Age Interaction – P-value** |  | 0.1043 |  | 0.6394 |  | **<0.0001**** |
| **NS(DBP, 3)1* Age 50-60** | -0.012 (-0.108, 0.085) | 0.8095 | -0.026 (-0.117, 0.064) | 0.5688 | 0.108 (0.052, 0.164) | **0.0002**** |
| **NS(DBP, 3)2* Age 50-60** | -0.201 (-0.642, 0.239) | 0.3706 | -0.129 (-0.55, 0.292) | 0.5483 | 0.105 (-0.148, 0.358) | 0.4142 |
| **NS(DBP, 3)3* Age 50-60** | -0.039 (-0.238, 0.16) | 0.6992 | -0.059 (-0.262, 0.143) | 0.5656 | 0.046 (-0.065, 0.158) | 0.4153 |
| **NS(DBP, 3)1* Age 60-70** | 0.091 (0.001, 0.182) | 0.0475 | -0.042 (-0.133, 0.05) | 0.3709 | 0.162 (0.109, 0.215) | **<0.0001**** |
| **NS(DBP, 3)2* Age 60-70** | 0.378 (-0.038, 0.794) | 0.0746 | 0.003 (-0.422, 0.428) | 0.9892 | 0.324 (0.084, 0.565) | **0.0082*** |
| **NS(DBP, 3)3* Age 60-70** | 0.14 (-0.055, 0.335) | 0.1584 | 0.087 (-0.122, 0.295) | 0.4155 | 0.111 (0.001, 0.222) | 0.0485 |

**Supplementary table 10:** Individual SBP/DBP natural splines terms and linear terms for the three main cognitive outcomes in a fully adjusted model, including an interaction between age (as a three-level variable) and the BP term. Those aged 40 – 50 were the comparison group. The table shows the overall significance of the interaction between age and the BP term by comparing fits of models with and without the interaction term. Analyses were adjusted for age (as a three level variable), sex, the Townsend deprivation index, education, cardiovascular disease history, diabetes status, smoking status, current physical activity, current alcohol use frequency, BMI and history of hypertension/antihypertensive use.

## Supplementary table 11: Individual Natural Splines Terms (SBP and DBP) including interaction with sex, Fully adjusted analysis

|  | **Fluid Intelligence** | | **Attention** | | **Reaction Time** | |
| --- | --- | --- | --- | --- | --- | --- |
| **SBP** | | | | | | |
|  | **B (95%CI)** | **P value** | **B (95%CI)** | **P value** | **B (95%CI)** | **P value** |
| **Sex** | 0.05 (-0.089, 0.188) | 0.4828 | -0.001 (-0.142, 0.14) | 0.9907 | -0.13 (-0.211, -0.048) | **0.0018*** |
| **NS(SBP, 3)1** | -0.048 (-0.097, 0.001) | 0.0529 | 0.078 (0.029, 0.128) | **0.0019*** | 0.158 (0.129, 0.187) | **<0.0001**** |
| **NS(SBP, 3)2** | 0.007 (-0.199, 0.214) | 0.9453 | 0.397 (0.185, 0.608) | **<0.0001**** | 0.424 (0.303, 0.545) | **<0.0001**** |
| **NS(SBP, 3)3** | -0.128 (-0.206, -0.05) | **0.0013*** | 0.006 (-0.077, 0.09) | 0.8815 | 0.075 (0.03, 0.121) | 0.0011 |
| **Sex Interaction – P-value** |  | **0.0031 **** |  | 0.1191 |  | **<0.0001**** |
| **NS(SBP, 3)1* Sex** | -0.028 (-0.095, 0.039) | 0.4127 | 0.065 (-0.003, 0.133) | 0.0623 | 0.204 (0.164, 0.243) | **<0.0001**** |
| **NS(SBP, 3)2* Sex** | -0.01 (-0.301, 0.28) | 0.9437 | 0.093 (-0.204, 0.391) | 0.5389 | 0.471 (0.3, 0.641) | **<0.0001**** |
| **NS(SBP, 3)3* Sex** | -0.064 (-0.173, 0.045) | 0.2482 | -0.02 (-0.138, 0.097) | 0.7366 | 0.156 (0.092, 0.219) | **<0.0001**** |
| **DBP** | | | | | | |
|  | **B (95%CI)** | **P value** | **B (95%CI)** | **P value** | **B (95%CI)** | **P value** |
| **Sex** | 0.123 (0.001, 0.245) | 0.0473 | 0.014 (-0.107, 0.135) | 0.8204 | 0.073 (0.002, 0.143) | 0.0449 |
| **NS(DBP, 3)1** | -0.125 (-0.167, -0.082) | **<0.0001**** | 0.022 (-0.02, 0.064) | 0.3094 | 0.048 (0.023, 0.072) | **0.0002**** |
| **NS(DBP, 3)2** | -0.141 (-0.326, 0.044) | 0.1361 | 0.219 (0.033, 0.405) | 0.0208 | 0.096 (-0.012, 0.204) | 0.0807 |
| **NS(DBP, 3)3** | -0.13 (-0.21, -0.05) | **0.0014*** | -0.009 (-0.092, 0.074) | 0.834 | -0.016 (-0.062, 0.029) | 0.4838 |
| **Sex Interaction – P-value** |  | **0.0170 *** |  | 0.7205 |  | **<0.0001**** |
| **NS(DBP, 3)1*Sex** | -0.053 (-0.112, 0.006) | 0.0761 | 0.026 (-0.033, 0.084) | 0.3891 | 0.082 (0.047, 0.117) | **<0.0001**** |
| **NS(DBP, 3)2*Sex** | -0.152 (-0.413, 0.109) | 0.2545 | 0.12 (-0.142, 0.383) | 0.3678 | 0.074 (-0.078, 0.226) | 0.3413 |
| **NS(DBP, 3)3*Sex** | -0.094 (-0.206, 0.017) | 0.0974 | 0.055 (-0.061, 0.171) | 0.3536 | 0.045 (-0.019, 0.109) | 0.1673 |

**Supplementary table 11:** Individual SBP/DBP natural splines terms and linear terms for the three main cognitive outcomes in a fully adjusted model, including an interaction between sex and the BP term. Females were the comparison group. The table shows the overall significance of the interaction between age and the BP term by comparing fits of models with and without the interaction term. Analyses were adjusted for age, age^2^, sex, the Townsend deprivation index, education, cardiovascular disease history, diabetes status, smoking status, current physical activity, current alcohol use frequency, BMI and history of hypertension/antihypertensive use.

## Supplementary table 12: Individual Natural Splines Terms (SBP and DBP) including interaction with hypertension history/antihypertensive use, Fully adjusted analysis

|  | **Fluid Intelligence** | | **Attention** | | **Reaction Time** | |
| --- | --- | --- | --- | --- | --- | --- |
| **SBP** | | | | | | |
|  | **B (95%CI)** | **P value** | **B (95%CI)** | **P value** | **B (95%CI)** | **P value** |
| **“Healthy Controls”** | - | - | **-** | **-** | **-** | **-** |
| **Treated Hypertension** | 0.023 (-0.314, 0.36) | 0.8924 | -0.218 (-0.636, 0.2) | 0.3063 | -0.25 (-0.444, -0.055) | **0.012*** |
| **Untreated Hypertension** | -0.357 (-0.859, 0.145) | 0.163 | -0.121 (-0.664, 0.423) | 0.6639 | -0.386 (-0.654, -0.119) | 0.0046* |
| **NS(SBP, 3)1** | -0.039 (-0.082, 0.004) | 0.0724 | 0.051 (0.01, 0.092) | **0.0149*** | 0.064 (0.039, 0.088) | **<0.0001**** |
| **NS(SBP, 3)2** | 0.068 (-0.125, 0.261) | 0.4892 | 0.314 (0.125, 0.502) | **0.0011*** | 0.108 (-0.005, 0.22) | 0.06 |
| **NS(SBP, 3)3** | -0.131 (-0.225, -0.037) | **0.0065*** | -0.005 (-0.104, 0.095) | 0.9251 | -0.021 (-0.076, 0.033) | 0.4409 |
| **HT/AHT use Interaction – P-value** |  | **0.0008**** |  | 0.7116 |  | **<0.0001**** |
| **NS(SBP, 3)1* Treated Hypertension** | -0.045 (-0.217, 0.126) | 0.6041 | 0.054 (-0.158, 0.265) | 0.6194 | 0.114 (0.015, 0.213) | 0.0235 |
| **NS(SBP, 3)2* Treated Hypertension** | -0.09 (-0.779, 0.599) | 0.7979 | 0.286 (-0.566, 1.137) | 0.5109 | 0.388 (-0.01, 0.786) | 0.056 |
| **NS(SBP, 3)3* Treated Hypertension** | 0.001 (-0.228, 0.23) | 0.994 | 0.04 (-0.241, 0.321) | 0.7799 | 0.103 (-0.03, 0.236) | 0.1276 |
| **NS(SBP, 3)1* Untreated Hypertension** | 0.298 (0.035, 0.561) | 0.0264 | 0.099 (-0.185, 0.384) | 0.4934 | 0.266 (0.126, 0.406) | **0.0002**** |
| **NS(SBP, 3)2* Untreated Hypertension** | 0.564 (-0.439, 1.567) | 0.2707 | 0.142 (-0.945, 1.229) | 0.7977 | 0.659 (0.124, 1.195) | **0.0159*** |
| **NS(SBP, 3)3* Untreated Hypertension** | 0.2 (-0.091, 0.49) | 0.1778 | 0.021 (-0.297, 0.338) | 0.8989 | 0.21 (0.052, 0.367) | **0.0091*** |
| **DBP** | | | | | | |
|  | **B (95%CI)** | **P value** | **B (95%CI)** | **P value** | **B (95%CI)** | **P value** |
| **No Hypertension** | - | - | - | - | - | - |
| **Treated Hypertension** | -0.118 (-0.357, 0.121) | 0.3319 | 0.078 (-0.224, 0.381) | 0.6123 | -0.082 (-0.224, 0.06) | 0.2559 |
| **Untreated Hypertension** | -0.478 (-0.886, -0.07) | 0.0217 | -0.3 (-0.766, 0.167) | 0.2085 | -0.002 (-0.233, 0.228) | 0.9849 |
| **NS(DBP, 3)1** | -0.126 (-0.168, -0.084) | **<0.0001**** | 0.015 (-0.025, 0.055) | 0.4714 | 0.017 (-0.008, 0.041) | 0.1852 |
| **NS(DBP, 3)2** | -0.097 (-0.29, 0.097) | 0.3273 | 0.175 (-0.012, 0.361) | 0.0669 | 0.048 (-0.064, 0.161) | 0.4015 |
| **NS(DBP, 3)3** | -0.115 (-0.216, -0.013) | 0.0277 | -0.036 (-0.139, 0.066) | 0.4888 | -0.04 (-0.098, 0.018) | 0.1802 |
| **HT/AHT use Interaction – P-value** |  | **<0.0001**** |  | 0.4932 |  | **0.0003**** |
| **NS(DBP, 3)1* Treated Hypertension** | 0.055 (-0.063, 0.174) | 0.3617 | -0.054 (-0.203, 0.095) | 0.4769 | -0.111 (-0.189, -0.034) | **0.0048*** |
| **NS(DBP, 3)2* Treated Hypertension** | 0.096 (-0.415, 0.608) | 0.7124 | -0.289 (-0.93, 0.352) | 0.377 | 0.056 (-0.014, 0.126) | 0.116 |
| **NS(DBP, 3)3* Treated Hypertension** | -0.1 (-0.316, 0.117) | 0.3666 | -0.005 (-0.257, 0.248) | 0.9717 | 0.055 (-0.249, 0.358) | 0.7241 |
| **NS(DBP, 3)1* Untreated Hypertension** | 0.352 (0.14, 0.564) | **0.0011*** | 0.107 (-0.136, 0.35) | 0.3883 | 0.043 (-0.084, 0.17) | 0.5071 |
| **NS(DBP, 3)2* Untreated Hypertension** | 0.803 (-0.026, 1.631) | 0.0577 | 0.586 (-0.359, 1.532) | 0.2241 | 0.061 (-0.059, 0.181) | 0.3167 |
| **NS(DBP, 3)3* Untreated Hypertension** | 0.238 (-0.018, 0.494) | 0.0682 | 0.175 (-0.112, 0.462) | 0.2329 | -0.092 (-0.56, 0.377) | 0.7016 |

**Supplementary table 12:** Individual SBP/DBP natural splines terms and linear terms for the three main cognitive outcomes in a fully adjusted model, including an interaction between sex and the hypertension history/antihypertensive use term (three level variable). “Healthy Controls”, those without a history of hypertension or taking an antihypertensive, were the comparison group. The table shows the overall significance of the interaction between age and the BP term by comparing fits of models with and without the interaction term. Analyses were adjusted for age, age^2^, sex, the Townsend deprivation index, education, cardiovascular disease history, diabetes status, smoking status, current physical activity, current alcohol use frequency, BMI and history of hypertension/antihypertensive use.

## Supplementary table 13: Individual Splines terms SBP/DBP, linear terms and antihypertensive use examining relationships with Cognitive baseline scores and trajectories over time, Fully Adjusted Analysis

|  | **Fluid Intelligence Score (n = 16,358)** | | **Attention Score (n = 13,286)** | | **Reaction Time (n = 52,222)** | |
| --- | --- | --- | --- | --- | --- | --- |
|  | **B (95%CI)** | **p** | **B (95%CI)** | **p** | **B (95%CI)** | **p** |
| **SBP** | | | | | | |
| **Time in Study (TIS)** | 0 (-0.073, 0.074) | 0.9982 | 0.412 (0.351, 0.474) | **<0.0001**** | -0.34 (-0.388, -0.293) | **<0.0001**** |
| **Treated Hypertension** | -0.019 (-0.064, 0.026) | 0.406 | -0.053 (-0.092, -0.014) | 0.0078 | -0.059 (-0.083, -0.035) | **<0.0001**** |
| **Untreated Hypertension** | 0.036 (-0.007, 0.079) | 0.1019 | 0.013 (-0.023, 0.049) | 0.4805 | -0.028 (-0.05, -0.006) | 0.0121 |
| **Treated Hypertension* Time in Study (TIS)** | -0.031 (-0.057, -0.005) | 0.0184 | -0.028 (-0.048, -0.008) | **0.0066*** | -0.02 (-0.034, -0.005) | **0.0105*** |
| **Untreated Hypertension*Time in Study (TIS)** | 0.022 (-0.005, 0.049) | 0.109 | 0.011 (-0.009, 0.03) | 0.2943 | -0.01 (-0.025, 0.004) | 0.1644 |
|  |  |  |  |  |  |  |
| **ns(SBP), 1** | -0.083 (-0.158, -0.008) | 0.0301 | 0.101 (0.035, 0.167) | **0.0026*** | 0.115 (0.074, 0.157) | **<0.0001**** |
| **ns(SBP), 2** | -0.12 (-0.404, 0.163) | 0.4057 | 0.16 (-0.098, 0.418) | 0.2248 | 0.166 (0.003, 0.33) | 0.0466 |
| **ns(SBP), 3** | -0.126 (-0.306, 0.054) | 0.171 | -0.044 (-0.193, 0.106) | 0.5666 | 0.048 (-0.046, 0.141) | 0.3157 |
|  |  |  |  |  |  |  |
| **Overall P-Value for effect over time** |  | 0.1098 |  | **<0.0001**** |  | 0.1499 |
| **ns(SBP), 1*TIS** | -0.02 (-0.063, 0.024) | 0.3777 | -0.085 (-0.119, -0.051) | **<0.0001**** | 0.008 (-0.018, 0.034) | 0.5506 |
| **ns(SBP), 2*TIS** | -0.083 (-0.255, 0.089) | 0.343 | -0.195 (-0.334, -0.055) | **0.0064*** | 0.088 (-0.019, 0.196) | 0.1073 |
| **ns(SBP), 3*TIS** | -0.094 (-0.207, 0.018) | 0.1009 | -0.053 (-0.135, 0.029) | 0.2033 | 0.011 (-0.053, 0.074) | 0.7437 |
| **DBP** | | | | | | |
| **Time in Study (TIS)** | 0.012 (-0.106, 0.13) | 0.843 | 0.318 (0.257, 0.38) | **<0.0001**** | -0.344 (-0.412, -0.277) | **<0.0001**** |
| **Treated Hypertension** | -0.016 (-0.061, 0.029) | 0.4848 | -0.049 (-0.088, -0.01) | **0.0133*** | -0.054 (-0.077, -0.03) | **<0.0001**** |
| **Untreated Hypertension** | 0.045 (0.002, 0.088) | 0.0422 | 0.022 (-0.014, 0.059) | 0.2226 | -0.02 (-0.042, 0.002) | 0.0697 |
| **Treated Hypertension* Time in Study (TIS)** | -0.037 (-0.062, -0.011) | **0.0054*** | -0.035 (-0.055, -0.015) | **0.0006**** | -0.022 (-0.036, -0.007) | **0.0041*** |
| **Untreated Hypertension*Time in Study (TIS)** | 0.015 (-0.011, 0.042) | 0.2608 | 0.003 (-0.017, 0.022) | 0.8048 | -0.014 (-0.029, 0.001) | 0.064 |
|  |  |  |  |  |  |  |
| **ns(DBP), 1** | -0.122 (-0.216, -0.028) | 0.0109 | 0.016 (-0.046, 0.079) | 0.6053 | 0.056 (0.006, 0.106) | 0.0268 |
| **ns(DBP), 2** | -0.374 (-0.786, 0.039) | 0.076 | -0.265 (-0.517, -0.014) | 0.0387 | 0.094 (-0.127, 0.315) | 0.4043 |
| **ns(DBP), 3** | -0.342 (-0.545, -0.139) | **0.0009**** | -0.209 (-0.347, -0.072) | **0.0028*** | 0.028 (-0.078, 0.134) | 0.6055 |
|  |  |  |  |  |  |  |
| **Overall P-Value for effect over time** |  | 0.6393 |  | 0.4263 |  | 0.3434 |
| **ns(DBP), 1*TIS** | -0.002 (-0.059, 0.056) | 0.9588 | -0.023 (-0.056, 0.01) | 0.174 | 0.012 (-0.02, 0.045) | 0.4635 |
| **ns(DBP), 2*TIS** | -0.104 (-0.363, 0.155) | 0.4313 | 0.006 (-0.133, 0.144) | 0.9343 | 0.122 (-0.026, 0.27) | 0.107 |
| **ns(DBP), 3*TIS** | -0.081 (-0.207, 0.045) | 0.2072 | 0.013 (-0.063, 0.088) | 0.7405 | 0.065 (-0.006, 0.135) | 0.0727 |

**Supplementary table 13:** Analysis of SBP/DBP and the three cognitive outcomes using mixed effects models including a natural splines term for BP and interaction with time in study. All models were adjusted for age, age^2^, sex, education, townsend index of deprivation, cardiovascular disease history, diabetes status, smoking status, current physical activity, current alcohol use frequency, BMI and history of hypertension/antihypertensive use.

There was an interaction between Time in Study (TIS) (centered at mean time in study) and the baseline BP.

The SBP/DBP P value for effect over time shows the significance of difference in model fits between the models with and without the TIS*BP natural splines term.

*p<0.017.

**p<0.001.

## Supplementary table 14: Association between baseline hypertension status/antihypertensive use and the three cognitive outcomes at baseline and over time

|  | **Fluid Intelligence (n=143,979)** | | **Attention (n=91,404)** | | **Reaction Time (n=427,396)** | |
| --- | --- | --- | --- | --- | --- | --- |
| **Outcomes** | **B (95%CI)** | **P value** | **B (95%CI)** | **P value** | **B (95%CI)** | **P value** |
| **Treated Hypertension** | -0.035 (-0.051, -0.02) | **<0.0001**** | -0.074 (-0.091, -0.057) | **<0.0001**** | -0.052 (-0.061, -0.043) | **<0.0001**** |
| **Untreated Hypertension** | -0.034 (-0.049, -0.018) | **<0.0001**** | -0.025 (-0.041, -0.009) | 0.0022* | -0.031 (-0.04, -0.022) | **<0.0001**** |
| **Longitudinal Analysis** | | | | | | |
| **Treated Hypertension* Time in Study (TIS)** | -0.031 (-0.057, -0.005) | 0.0184 | -0.028 (-0.048, -0.008) | **0.0066*** | -0.02 (-0.034, -0.005) | **0.0105*** |
| **Untreated Hypertension*Time in Study (TIS)** | 0.022 (-0.005, 0.049) | 0.109 | 0.011 (-0.009, 0.03) | 0.2943 | -0.01 (-0.025, 0.004) | 0.1644 |

**Supplementary Table 14 –** Association between baseline hypertension status/antihypertensive use and the three cognitive outcomes at baseline and over time (with the time interaction). Time in Study was measured in intervals of 5 years and as such a B of -0.028 indicates a poorer cognitive trajectory of 0.028 standard deviations over 5 years.

*<0.017

**<0.001

## Supplementary table 15: Non-linear relationship between SBP/DBP and TMTA, TMTB and DSS, Fully Adjusted Analysis

| **SBP** | | | |
| --- | --- | --- | --- |
|  | **Predicted TMTA score (SD) (95%CI) (n = 93563)** | **Predicted TMTB score (SD) (95%CI) (n = 93561)** | **Predicted DSS score (SD) (95%CI) (n = 106184)** |
| **p-value** | **<0.0001**** | **<0.0001**** | **<0.0001**** |
| **ideal BP (mmHg) (95%CI)** | 127.5 (123.0, 130.5) | 127 (121.5, 130.5) | 129 (126.5, 134.5) |
| **100** | -0.043 (-0.073, -0.012) | -0.03 (-0.059, -0.001) | -0.05 (-0.076, -0.023) |
| **120** | 0.015 (0.007, 0.023) | 0.013 (0.006, 0.021) | 0.008 (0.001, 0.015) |
| **140** | 0.007 (0.001, 0.012) | 0.005 (0, 0.011) | 0.007 (0.003, 0.012) |
| **160** | -0.027 (-0.038, -0.016) | -0.024 (-0.035, -0.014) | -0.016 (-0.026, -0.007) |
| **180** | -0.057 (-0.081, -0.032) | -0.053 (-0.076, -0.029) | -0.037 (-0.058, -0.016) |
| **DBP** | | | |
|  | **Predicted TMTA score (SD) (95%CI) (n = 93563)** | **Predicted TMTB score (SD) (95%CI) (n = 93561)** | **Predicted DSS score (SD) (95%CI) (n = 106184)** |
| **p-value** | **0.0001**** | **0.0001**** | **0.0005**** |
| **ideal BP (mmHg) (95%CI)** | 75 (62.9, 78.5) | 71.5 (46.5, 122.5) | 76.5 (74.5, 122.5) |
| **60** | -0.017 (-0.047, 0.012) | 0.003 (-0.026, 0.031) | -0.036 (-0.062, -0.011) |
| **70** | 0.011 (0.002, 0.02) | 0.016 (0.007, 0.025) | 0.003 (-0.005, 0.011) |
| **80** | 0.011 (0.005, 0.016) | 0.008 (0.002, 0.013) | 0.01 (0.005, 0.015) |
| **90** | -0.012 (-0.021, -0.003) | -0.015 (-0.023, -0.007) | -0.007 (-0.015, 0.001) |
| **100** | -0.032 (-0.048, -0.017) | -0.029 (-0.044, -0.015) | -0.019 (-0.032, -0.006) |

**Supplementary table 15:** Association between baseline SBP/DBP and the three cognitive outcomes that make up the composite attention cognitive scores. The table shows the significance of the overall natural splines term and whether the natural splines term produced a significantly better model fit compare to a linear term for BP (p-value vs linear). All natural splines terms included three degrees of freedom. The table shows the predicted cognitive scores at various points along the range of SBPs and DBPs as well as the 95% confidence intervals at those points**.** Analyses were adjusted for age, age^2^, sex, the Townsend deprivation index, education, cardiovascular disease history, diabetes status, smoking status, current physical activity, current alcohol use frequency, BMI and history of hypertension/antihypertensive use.

TMTA - Trail Making Test A; TMTB – Trail Making Test B; DSS – Digit Symbol Substitution test.

*<0.017

**<0.001

## Supplementary table 16: Pulse Pressure and cognitive scores, Fully Adjusted Analysis

| **PP** | | | |
| --- | --- | --- | --- |
|  | **Predicted Fluid Intelligence (SD) (95%CI) (n = 143,979)** | **Predicted Attention Score (SD) (95%CI) (n = 91,404)** | **Predicted Reaction Time (SD) (95%CI) (n = 427,396)** |
| **p-value** | **<0.0001**** | **<0.0001**** | **<0.0001**** |
| **ideal PP (mmHg) (95%CI)** | 50 (47, 55.5) | 50.5 (47.5, 57.5) | 64.5 (57.5, 69) |
| **35** | -0.015 (-0.029, 0) | -0.017 (-0.031, -0.003) | -0.038 (-0.047, -0.03) |
| **45** | 0.009 (0.003, 0.015) | 0.006 (0.001, 0.012) | -0.007 (-0.01, -0.003) |
| **55** | 0.009 (0.006, 0.013) | 0.008 (0.004, 0.012) | 0.009 (0.007, 0.012) |
| **65** | -0.003 (-0.01, 0.004) | -0.002 (-0.01, 0.005) | 0.013 (0.009, 0.017) |
| **75** | -0.018 (-0.027, -0.009) | -0.015 (-0.025, -0.006) | 0.009 (0.004, 0.014) |

**Supplementary table 16:** Association between baseline Pulse Pressure and the three cognitive outcomes that make up the composite attention cognitive scores. The table shows the significance of the overall natural splines term and whether the natural splines term produced a significantly better model fit compare to a linear term for BP (p-value vs linear). All natural splines terms included three degrees of freedom. The table shows the predicted cognitive scores at various points along the range of SBPs and DBPs as well as the 95% confidence intervals at those points**.** Analyses were adjusted for age, age^2^, sex, the Townsend deprivation index, education, cardiovascular disease history, diabetes status, smoking status, current physical activity, current alcohol use frequency, BMI and history of hypertension/antihypertensive use.

*<0.017

**<0.001

## Supplementary table 17: Individual Natural Splines Terms, Pulse Pressure, Fully-Adjusted Analysis

|  | **Fluid Intelligence** | | **Attention** | | **Reaction Time** | |
| --- | --- | --- | --- | --- | --- | --- |
|  | **B (95%CI)** | **P value** | **B (95%CI)** | **P value** | **B (95%CI)** | **P value** |
| **p-value v null** |  | **<0.0001**** |  | **<0.0001**** |  | **<0.0001**** |
| **p-value v linear** |  | **<0.0001**** |  | **0.0001**** |  | **<0.0001**** |
| **NS(PP, 3)1** | 0.091 (0.03, 0.152) | **0.0034*** | 0.094 (0.034, 0.153) | **0.0022*** | 0.151 (0.116, 0.186) | **<0.0001**** |
| **NS(PP, 3)2** | 0.33 (0.074, 0.585) | **0.0114*** | 0.328 (0.072, 0.583) | **0.0119*** | 0.374 (0.226, 0.521) | **<0.0001**** |
| **NS(PP, 3)3** | -0.003 (-0.086, 0.08) | 0.9505 | 0.009 (-0.079, 0.098) | 0.8403 | 0.068 (0.02, 0.117) | **0.0055*** |

**Supplementary table 17:** Individual PP natural splines terms for the three main cognitive outcomes (Fluid Intelligence, Attention and Reaction time). All natural splines terms included three degrees of freedom. Analyses were adjusted for age, age^2^, sex, the Townsend deprivation index, education, cardiovascular disease history, diabetes status, smoking status, current physical activity, current alcohol use frequency, BMI and history of hypertension/antihypertensive use.

*<0.017

**<0.001

## Supplementary table 18: Non-linear relationship between SBP/DBP and Cognitive outcomes, Partially Adjusted Analysis

| **SBP** | | | |
| --- | --- | --- | --- |
|  | **Predicted Fluid Intelligence (SD) (95%CI) (n = 145,113)** | **Predicted Attention Score (SD) (95%CI) (n = 91,727)** | **Predicted Reaction Time (SD) (95%CI) (n = 430,208)** |
| **p-value** | **<0.0001**** | **<0.0001**** | **<0.0001**** |
| **p-value v linear** | **0.0003**** | **<0.0001**** | **<0.0001**** |
| **ideal BP (mmHg) (95%CI)** | 121.5 (109.5, 125.5) | 128 (125.5, 130.5) | 150 (145, 155) |
| **100** | -0.001 (-0.025, 0.023) | -0.054 (-0.078, -0.031) | -0.049 (-0.063, -0.035) |
| **120** | 0.022 (0.016, 0.028) | 0.012 (0.006, 0.018) | -0.011 (-0.015, -0.007) |
| **140** | 0.003 (-0.001, 0.007) | 0.008 (0.003, 0.012) | 0.01 (0.007, 0.012) |
| **160** | -0.031 (-0.039, -0.023) | -0.023 (-0.032, -0.015) | 0.01 (0.005, 0.014) |
| **180** | -0.06 (-0.077, -0.043) | -0.048 (-0.067, -0.029) | -0.006 (-0.015, 0.004) |
| **DBP** | | | |
|  | **Predicted Fluid Intelligence (SD) (95%CI) (n = 145,113)** | **Predicted Attention Score (SD) (95%CI) (n = 91,727)** | **Predicted Reaction Time (SD) (95%CI) (n = 430,208** |
| **p-value** | **<0.0001**** | **<0.0001**** | **<0.0001**** |
| **p-value v linear** | 0.1355 | **0.0002**** | **0.0001**** |
| **ideal BP (mmHg) (95%CI)** | 65.5 (42.5, 72.5) | 76.5 (73.5, 122.5) | 89 (83.5, 97) |
| **60** | 0.025 (0.002, 0.049) | -0.031 (-0.053, -0.008) | -0.029 (-0.042, -0.015) |
| **70** | 0.026 (0.019, 0.032) | 0.005 (-0.002, 0.012) | -0.01 (-0.014, -0.006) |
| **80** | 0.008 (0.004, 0.012) | 0.01 (0.005, 0.014) | 0.003 (0, 0.005) |
| **90** | -0.02 (-0.026, -0.013) | -0.008 (-0.015, -0.002) | 0.007 (0.004, 0.011) |
| **100** | -0.039 (-0.05, -0.028) | -0.021 (-0.032, -0.009) | 0.001 (-0.005, 0.007) |

**Supplementary table 18:** Association between baseline SBP/DBP and the three main cognitive outcomes (Fluid Intelligence, Attention and Reaction time) in a partially adjusted model. The table shows the significance of the overall natural splines term and whether the natural splines term produced a significantly better model fit compare to a linear term for BP (p-value vs linear). All natural splines terms included three degrees of freedom. The table shows the predicted cognitive scores at various points along the range of SBPs and DBPs as well as the 95% confidence intervals at those points**.** Analyses were adjusted for age, age^2^, sex, the Townsend deprivation index and education.

*<0.017

**<0.001

## Supplementary table 19: Individual Natural Splines Terms and Linear effect of SBP/DBP on cognitive outcomes, Partially Adjusted Analysis

| **Outcomes** | **Fluid Intelligence (n = 145,113)** | | **Attention (n = 91,727)** | | **Reaction Time (n = 430,208)** | |
| --- | --- | --- | --- | --- | --- | --- |
| **SBP** | | | | | | |
|  | **B (95%CI)** | **P value** | **B (95%CI)** | **P value** | **B (95%CI)** | **P value** |
| **p-value v null** |  | **<0.0001**** |  | **<0.0001**** |  | **<0.0001**** |
| **p-value v linear** |  | **0.0003**** |  | **<0.0001**** |  | **<0.0001**** |
| **NS(SBP, 3)1** | -0.011 (-0.05, 0.028) | 0.5787 | 0.075 (0.037, 0.113) | **0.0001**** | 0.102 (0.08, 0.125) | **<0.0001**** |
| **NS(SBP, 3)2** | 0.121 (-0.049, 0.292) | 0.1637 | 0.384 (0.214, 0.553) | **<0.0001**** | 0.205 (0.105, 0.304) | **0.0001**** |
| **NS(SBP, 3)3** | -0.087 (-0.157, -0.016) | 0.0159* | -0.003 (-0.078, 0.073) | 0.9422 | 0.016 (-0.025, 0.056) | 0.4568 |
| **DBP** | | | | | | |
| **Outcomes** | **B (95%CI)** | **P value** | **B (95%CI)** | **P value** | **B (95%CI)** | **P value** |
| **p-value v null** |  | <0.0001** |  | **<0.0001**** |  | **<0.0001**** |
| **p-value v linear** |  | 0.1355 |  | **0.0002**** |  | **0.0001**** |
| **NS(DBP, 3)1** | -0.061 (-0.1, -0.022) | **0.0021*** | 0.043 (0.004, 0.081) | 0.0289 | 0.059 (0.037, 0.082) | **<0.0001**** |
| **NS(DBP, 3)2** | -0.015 (-0.191, 0.161) | 0.8665 | 0.26 (0.084, 0.436) | **0.0037*** | 0.11 (0.008, 0.212) | 0.0354 |
| **NS(DBP, 3)3** | -0.085 (-0.163, -0.008) | 0.0306 | 0.017 (-0.062, 0.097) | 0.6688 | 0.003 (-0.041, 0.047) | 0.9058 |

**Supplementary table 19:** Individual SBP/DBP natural splines terms for the three main cognitive outcomes (Fluid Intelligence, Attention and Reaction time) in a partially adjusted model. The table shows the significance of the overall natural splines term and whether the natural splines term produced a significantly better model fit compare to a linear term for BP (p-value vs linear). All natural splines terms included three degrees of freedom. Analyses were adjusted for age, age^2^, sex, the Townsend deprivation index and education.

*<0.017

**<0.001

## Supplementary table 20: Non-linear relationship between SBP/DBP and Cognitive outcomes, restricted analysis in those with complete cognitive scores

| **SBP** | | | |
| --- | --- | --- | --- |
|  | **Predicted Fluid Intelligence (SD) (95%CI) (n = 34,635)** | **Predicted Attention Score (SD) (95%CI) (n = 34,635)** | **Predicted Reaction Time (SD) (95%CI) (n = 34,635)** |
| **p-value** | **<0.0001**** | **0.0001**** | **0.0003**** |
| **ideal BP (mmHg) (95%CI)** | 118.5 (79, 128) | 126.5 (120, 133) | 148.5 (137, 157) |
| **100** | 0.008 (-0.041, 0.058) | -0.034 (-0.075, 0.006) | -0.064 (-0.113, -0.015) |
| **120** | 0.027 (0.014, 0.04) | 0.014 (0.004, 0.025) | -0.017 (-0.03, -0.005) |
| **140** | -0.002 (-0.011, 0.006) | 0.006 (-0.001, 0.013) | 0.018 (0.01, 0.026) |
| **160** | -0.039 (-0.056, -0.023) | -0.025 (-0.039, -0.011) | 0.015 (-0.002, 0.031) |
| **DBP** | | | |
|  | **Predicted Fluid Intelligence (SD) (95%CI) (n = 34,635)** | **Predicted Attention Score (SD) (95%CI) (n = 34,635)** | **Predicted Reaction Time (SD) (95%CI) (n = 34,635)** |
| **p-value** | **<0.0001**** | 0.0303 | 0.2749 |
| **ideal BP (mmHg) (95%CI)** | 48 (48, 71.5) | 74.5 (48, 122) | 85.5 (48, 122) |
| **60** | 0.063 (0.016, 0.111) | -0.012 (-0.051, 0.027) | -0.015 (-0.062, 0.032) |
| **70** | 0.042 (0.028, 0.057) | 0.01 (-0.002, 0.022) | -0.006 (-0.02, 0.009) |
| **80** | 0.007 (-0.002, 0.015) | 0.008 (0.002, 0.015) | 0.005 (-0.003, 0.013) |
| **90** | -0.033 (-0.047, -0.019) | -0.01 (-0.021, 0.001) | 0.006 (-0.008, 0.019) |
| **100** | -0.057 (-0.082, -0.033) | -0.027 (-0.048, -0.007) | -0.016 (-0.04, 0.008) |

**Supplementary table 20:** Association between baseline SBP/DBP and the three main cognitive outcomes (Fluid Intelligence, Attention and Reaction time) in a fully adjusted model that had been restricted to only those participants who had completed all cognitive tests at baseline. The table shows the significance of the overall natural splines term. All natural splines terms included three degrees of freedom. The table shows the predicted cognitive scores at various points along the range of SBPs and DBPs as well as the 95% confidence intervals at those points**.** Analyses were adjusted for age, age^2^, sex, the Townsend deprivation index and education.

## Supplementary Figure 1: Non-linear relationship between SBP and Cognitive Outcomes, Fully Adjusted Analysis


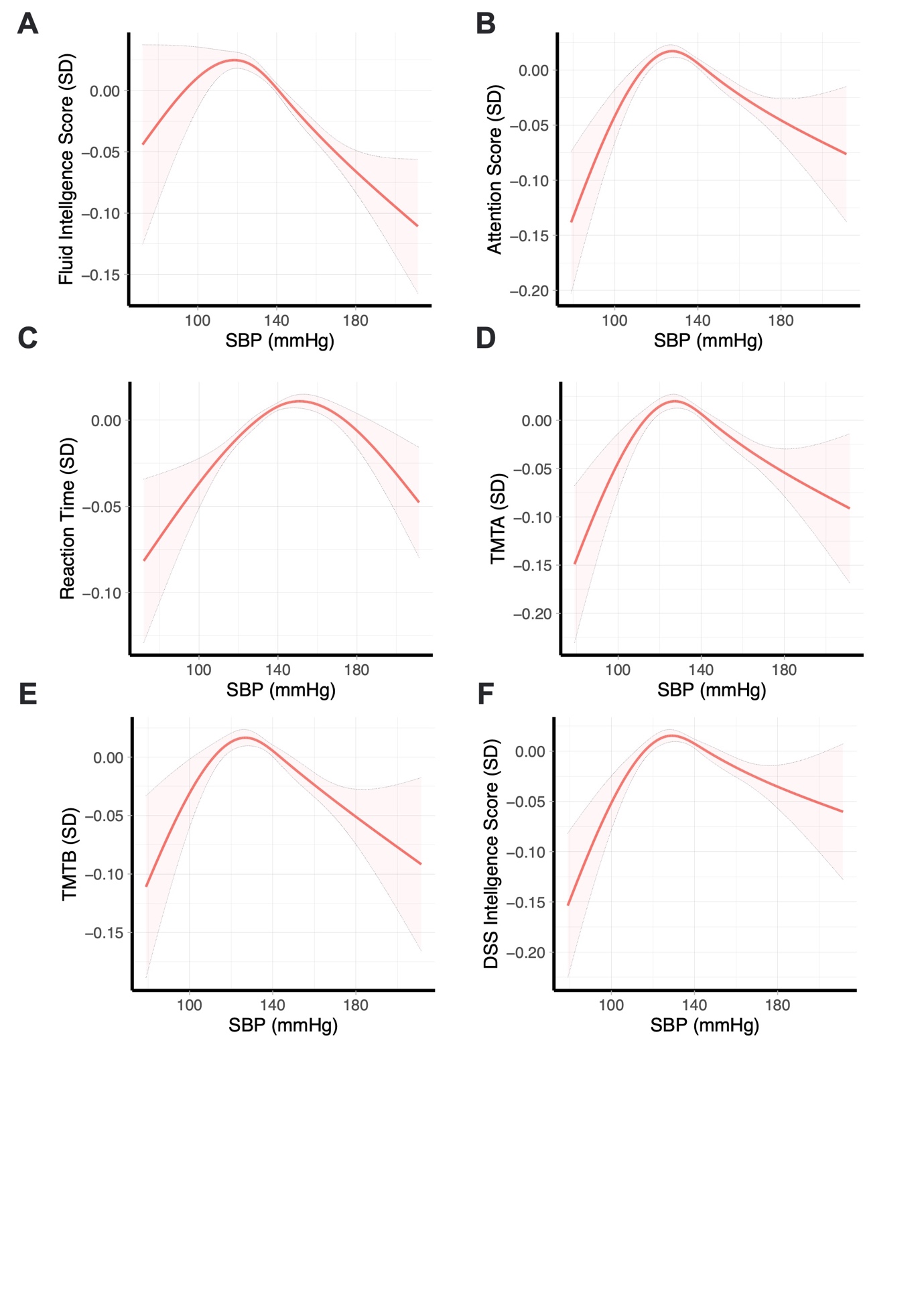


**Supplementary Figure 1:** Association between baseline SBP and the six standardised cognitive outcomes (three main outcomes and the three tests (TMTA, TMTB and DSS) comprising the attention score) in a fully adjusted analysis. All natural splines terms included three degrees of freedom. The figure shows the predicted cognitive scores at various points along the range of SBPs and the shaded area indicates 95% confidence intervals at those points**.** For each of the domains a higher score indicates better performance.

## Supplementary Figure 2: Non-linear relationship between DBP and Cognitive Outcomes, Fully Adjusted Analysis

**
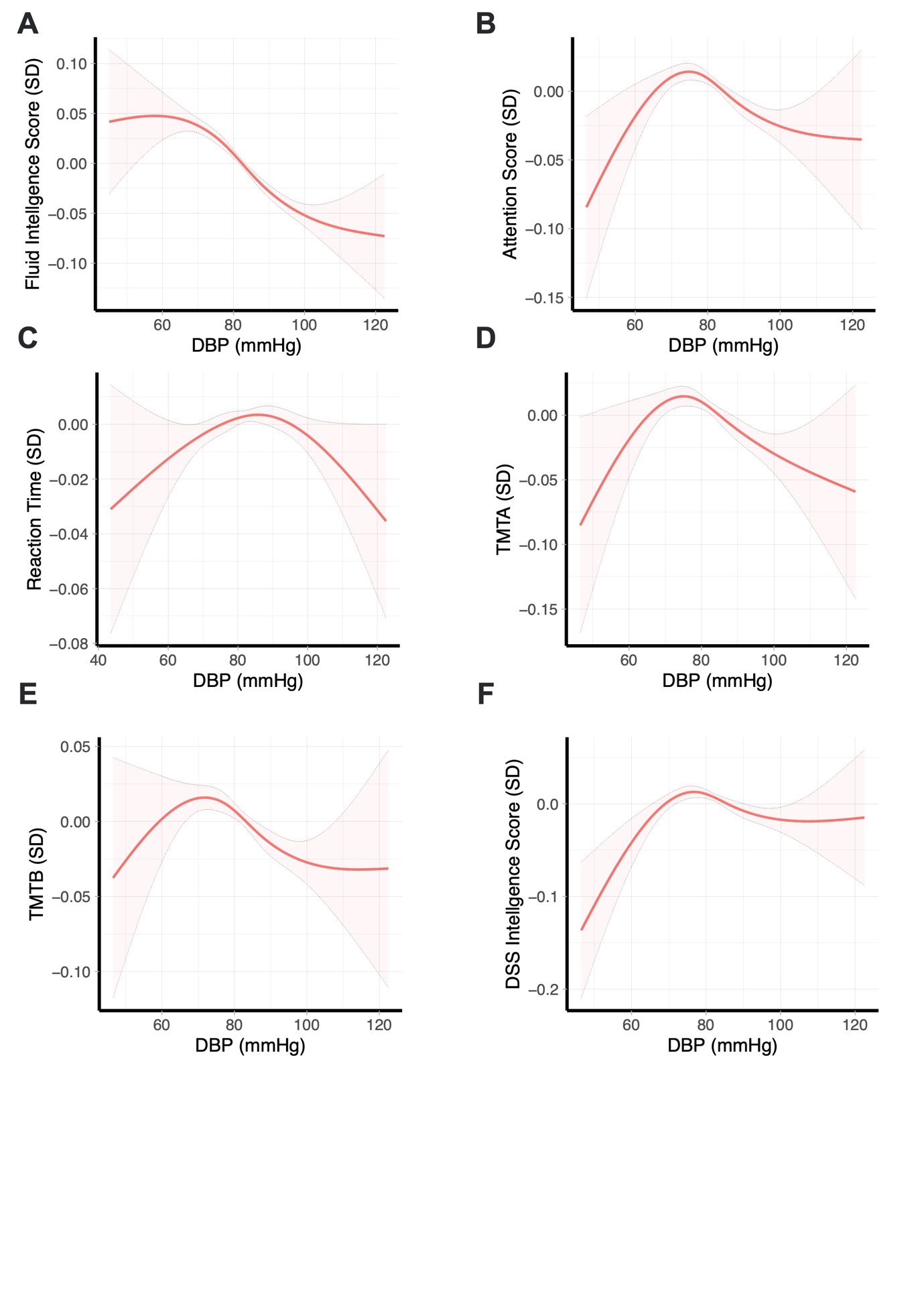
**

**Supplementary Figure 2 –** Association between baseline DBP and the six standardised cognitive outcomes (three main outcomes and the three tests (TMTA, TMTB and DSS) comprising the attention score) in a fully adjusted analysis. All natural splines terms included three degrees of freedom. The figure shows the predicted cognitive scores at various points along the range of DBPs and the shaded area indicates 95% confidence intervals at those points**.** For each of the domains a higher score indicates better performance.

## Supplementary Figure 3: Non-linear relationship between PP and Cognitive Outcomes, Fully Adjusted Analysis

**
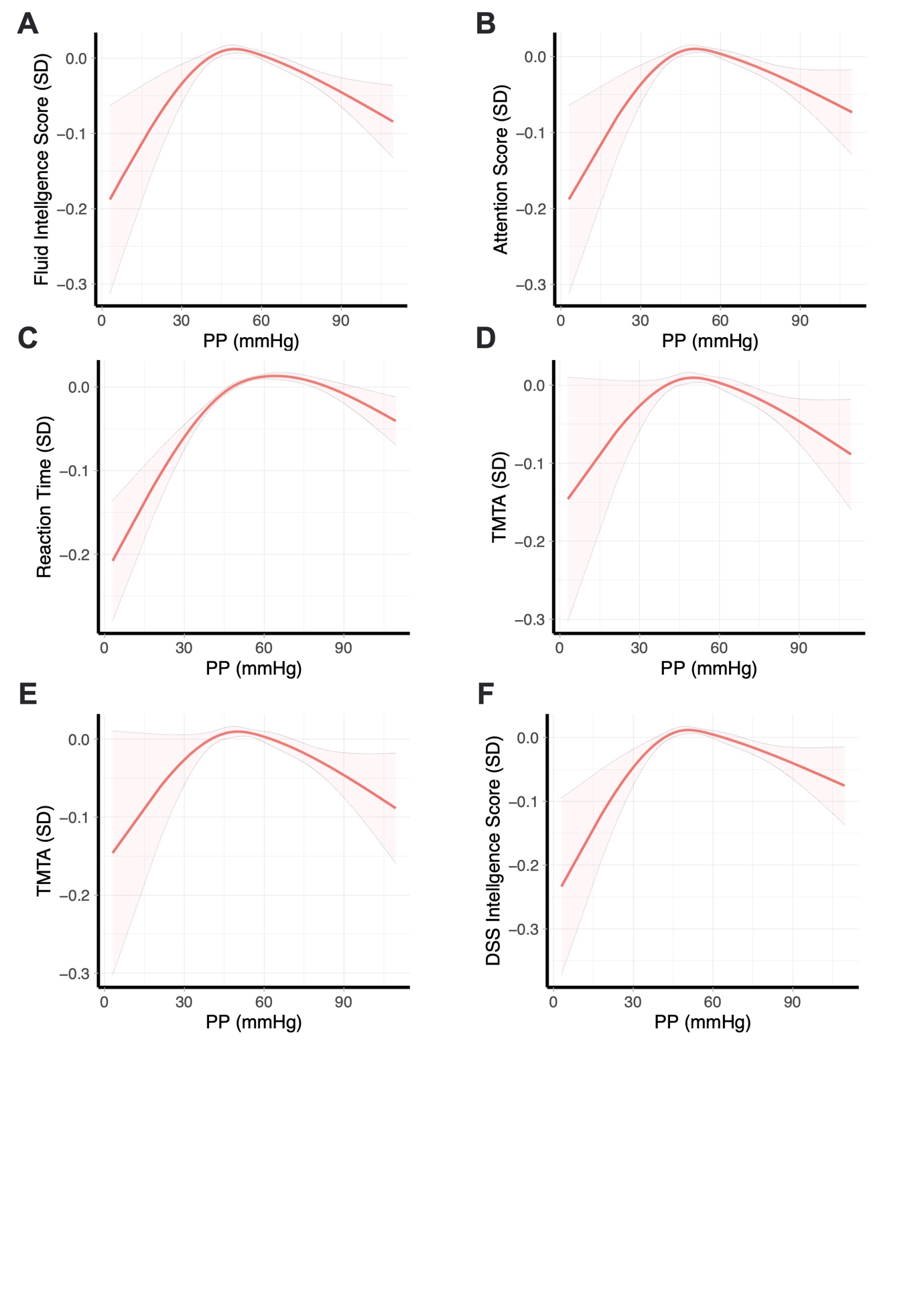
**

**Supplementary Figure 3 –** Association between baseline PP and the six standardised cognitive outcomes (three main outcomes and the three tests (TMTA, TMTB and DSS) comprising the attention score) in a fully adjusted analysis. All natural splines terms included three degrees of freedom. The figure shows the predicted cognitive scores at various points along the range of DBPs and the shaded area indicates 95% confidence intervals at those points**.** For each of the domains a higher score indicates better performance.

## Supplementary Figure 4: Non-linear relationship between SBP/DBP and Cognitive Outcomes, Partially Adjusted Analysis


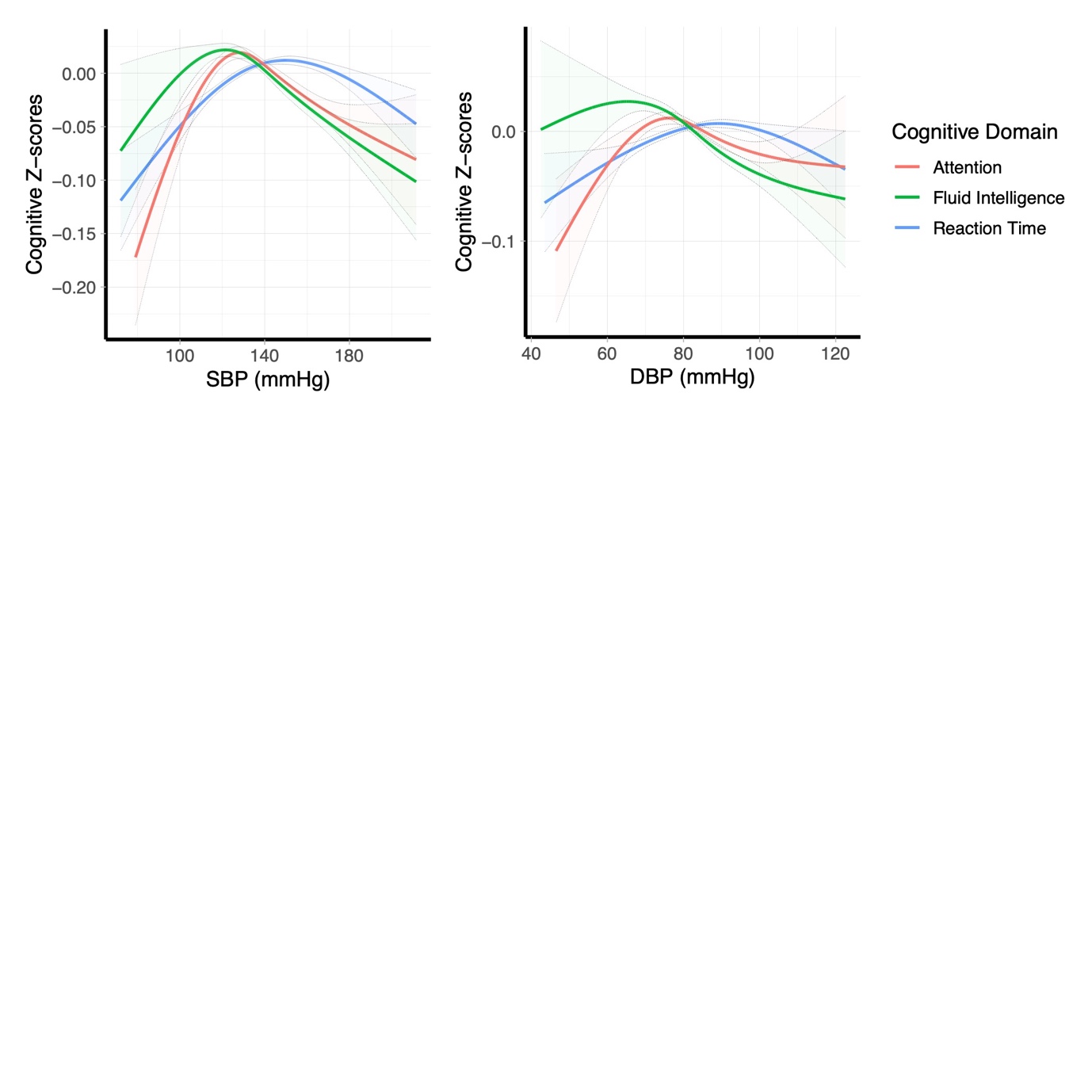


**Supplementary Figure 4 –** Association between baseline SBP/DBP and the three main cognitive outcomes in a partially adjusted analysis. All natural splines terms included three degrees of freedom. The figure shows the predicted cognitive scores at various points along the range of DBPs and the shaded area indicates 95% confidence intervals at those points**.** For each of the domains a higher score indicates better performance.

## Supplementary Figure 5: Non-linear relationship between SBP/DBP and Cognitive Outcomes, Analysis restricted to those with complete outcomes


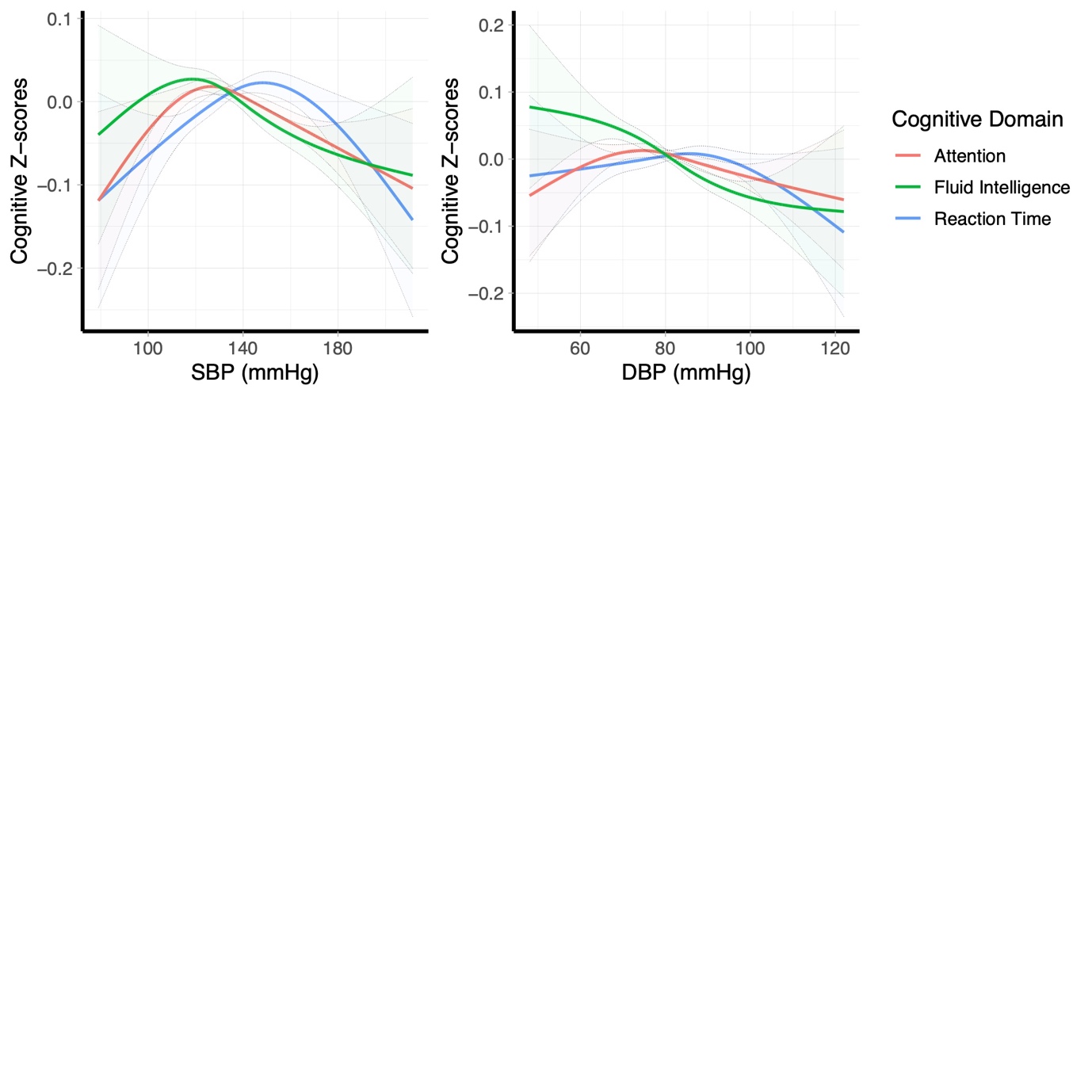


**Supplementary Figure 5 –** Association between baseline SBP/DBP and the three main cognitive outcomes in a fully adjusted analysis restricted to those participants who had all cognitive tests completed. All natural splines terms included three degrees of freedom. The figure shows the predicted cognitive scores at various points along the range of DBPs and the shaded area indicates 95% confidence intervals at those points**.** For each of the domains a higher score indicates better performance.
